# Long-term immunity and protection against SARS-CoV-2 XBB.1.5 following homologous and heterologous mRNA and MVA vaccination

**DOI:** 10.3389/fimmu.2026.1916109

**Published:** 2026-08-31

**Authors:** Patricia Pérez, Gloria Esteso, Isabel García-García, Sara Flores, Cristina Sánchez-Corzo, María A. Noriega, José M. Casasnovas, Mariano Esteban, Juan García-Arriaza

**Affiliations:** 1 Department of Molecular and Cellular Biology, Centro Nacional de Biotecnología (CNB), Consejo Superior de Investigaciones Científicas (CSIC), Madrid, Spain; 2 Centro de Investigación Biomédica en Red de Enfermedades Infecciosas (CIBERINFEC), Madrid, Spain; 3 Department of Genetics, Physiology and Microbiology, Faculty of Biological Sciences, Universidad Complutense de Madrid (UCM), Madrid, Spain; 4 Department of Macromolecular Structures, Centro Nacional de Biotecnología (CNB), Consejo Superior de Investigaciones Científicas (CSIC), Madrid, Spain

**Keywords:** durable immunity, mRNA vaccine, MVA, Omicron XBB.1.5, SARS-CoV-2

## Abstract

Although mRNA-based COVID-19 vaccines have demonstrated high efficacy, their widespread global use remains constrained by high production costs and cold-chain requirements. Modified vaccinia virus Ankara (MVA) is a highly attenuated and thermostable viral vector with low production costs, potent immunogenicity, and strong potential for global distribution. Here, we compared head-to-head the long-term immunogenicity and efficacy of an MVA-based vaccine candidate with an approved mRNA vaccine in K18-hACE2 mice, both expressing the SARS-CoV-2 Omicron XBB.1.5 spike (S) protein. Mice received by intramuscular route homologous (mRNA/mRNA and MVA/MVA), heterologous (mRNA/MVA), or single-dose MVA regimens. SARS-CoV-2-specific humoral and cellular responses were evaluated at 10 days and 9 months after the last vaccination, as well as antibody levels at intermediate time points, and protection was assessed following intranasal SARS-CoV-2 XBB.1.5 challenge at 9 months post-vaccination. Binding IgG antibodies against the XBB.1.5 S protein remained high throughout the 9-month period in all vaccinated groups, whereas neutralizing antibody titers peaked early after the last vaccination and progressively declined, converging across regimens over time. S-specific CD8^+^ T-cell responses were strongest in mRNA-containing regimens at day 10 after the last vaccination and, although they contracted over time, they remained detectable at 9 months after the last vaccination in all two-dose groups, with a trend toward enhanced persistence in the heterologous mRNA/MVA regimen. In contrast, S-specific CD4^+^ T-cell responses remained low across all groups. All two-dose regimens markedly reduced viral RNA levels and infectious viral titers in both the upper and lower respiratory tract following SARS-CoV-2 XBB.1.5 challenge. Transcriptomic analysis of lung tissue after virus challenge revealed reduced expression of genes associated with inflammatory myeloid responses, interferon signalling and cellular stress in vaccinated mice compared with infected controls. Distinct post-challenge lung transcriptional profiles were observed across vaccination regimens, with differential modulation of genes associated with humoral, innate, and cellular immune responses. Overall, our findings demonstrate that MVA-based vaccination induces durable immunity in mice and achieves long-term control of SARS-CoV-2 XBB.1.5 replication comparable to that of mRNA vaccination, supporting its use as an alternative and complementary vaccine platform against SARS-CoV-2 and other emerging respiratory viruses.

## Introduction

The rapid development of vaccines against SARS-CoV-2 using different technological platforms led to a dramatic reduction in the impact of the COVID-19 pandemic, saving millions of lives and preventing substantial economic losses for healthcare systems. However, none of these platforms is without limitations, and the lessons learned are essential for preparedness against the future emergence of SARS-CoV-2 variants, other coronaviruses, or viruses with pandemic potential.

Among these platforms, mRNA vaccines have been the most widely deployed and remain the predominant vaccines due to their high initial efficacy and rapid adaptability to emerging variants. Nevertheless, they present certain limitations, including stringent cold-chain requirements and waning humoral immunity that may necessitate periodic booster doses (1, 2).

The modified vaccinia virus Ankara (MVA), a highly attenuated poxvirus vector, represents another well-established vaccine platform due to its excellent safety profile, strong immunogenicity, and relatively low production cost. Several SARS-CoV-2 vaccines based on MVA vectors have reached phase 1 clinical trials, demonstrating favourable safety and the induction of specific antibody and T-cell responses, although none have yet been approved for widespread use (3, 4). Extensive preclinical studies have explored MVA-based SARS-CoV-2 vaccine candidates incorporating different antigen designs, including full-length native or prefusion-stabilized spike (S) proteins and receptor-binding domain (RBD)-based antigens, and have demonstrated robust humoral and cellular immune responses and protective efficacy across different animal models (5–10). Collectively, these studies support the versatility of MVA in both homologous and heterologous vaccination strategies. In our group, we have previously shown that MVA vectors expressing either the full-length native SARS-CoV-2 S protein (termed MVA-S) or a prefusion-stabilized S protein containing furin cleavage site mutations and three proline (3P) substitutions in the S2 region (A942P/K986P/V987P) [termed MVA-S(3P)] induce strong humoral and cellular immune responses and confer protective efficacy in mice (8, 9, 11–17), hamsters (18, 19), and rhesus macaques (20). Moreover, we demonstrated that MVA-S(3P) can induce robust systemic and mucosal antigen-specific humoral and T-cell responses against SARS-CoV-2, as well as protection against both disease and transmission, when used as a booster in K18-hACE2 mice and Syrian hamsters primed with Newcastle disease virus vector encoding a prefusion-stabilized S protein (16).

Heterologous vaccination regimens have therefore emerged as a promising strategy to overcome some of the limitations of homologous platforms. Multiple studies have demonstrated that these approaches are safe and effective against SARS-CoV-2 infection, can induce comparable or even higher S-specific antibody titers than homologous prime/boost schedules, display similar reactogenicity profiles, may elicit stronger immune responses in immunocompromised individuals, and can facilitate vaccination campaigns in regions facing fluctuating vaccine supplies (21). However, further studies are required to assess the long-term immunogenicity and efficacy of heterologous regimens. The potential of heterologous mRNA/MVA vaccination regimens has also been evaluated in challenge models, demonstrating balanced humoral and CD8^+^ T-cell memory responses, which are key to mitigating breakthrough infections in primed populations (7, 22). However, these studies did not comprehensively assess the durability and functional characteristics of vaccine-induced humoral and cellular immunity over extended follow-up periods. Furthermore, despite the substantial body of preclinical evidence supporting the immunogenicity and protective efficacy of both mRNA- and MVA-based SARS-CoV-2 vaccines, important gaps remain regarding their long-term durability and direct comparison under the same experimental conditions. Most studies have evaluated immune responses and protection up to 4–7 months post-vaccination (23, 24). However, direct longitudinal head-to-head comparisons of mRNA- and MVA-based platforms remain limited, particularly regarding the durability and breadth of antibody responses, Fc-mediated antibody functions, cellular immunity, and efficacy against antigenically divergent Omicron sublineages. To our knowledge, these parameters have not been comprehensively evaluated over a 9-month period using homologous and heterologous mRNA/MVA regimens targeting the Omicron XBB.1.5 sublineage and including a clinically approved variant-matched mRNA vaccine as comparator.

In this study, we address these gaps by directly comparing the commercial Comirnaty^®^ Omicron XBB.1.5 mRNA vaccine with an optimized MVA vector expressing a prefusion-stabilized XBB.1.5 S protein in K18-hACE2 mice. We evaluated homologous (mRNA/mRNA or MVA/MVA) and heterologous (mRNA/MVA) vaccination regimens, as well as a single-dose MVA strategy, and longitudinally assessed head-to-head humoral (binding IgG, neutralizing antibodies, and Fc functionality) and cellular (CD4^+^ and CD8^+^ T-cell) immune responses at 10 days and up to 9 months after the last vaccination. We further evaluated long-term protection against SARS-CoV-2 XBB.1.5 challenge at 9 months post-vaccination and characterized post-challenge lung transcriptional responses associated with the different vaccination regimens. Together, this study provides a comprehensive longitudinal comparison of homologous and heterologous mRNA/MVA vaccination strategies, offering insights into the durability of vaccine-induced immunity, antibody functionality, cellular immune responses, and long-term protection against the immune-evasive SARS-CoV-2 XBB.1.5 sublineage.

## Materials and methods

### Animals and ethics statement

Female K18-hACE2 transgenic mice (6–8 weeks old), which express the human ACE2 receptor, were purchased from the Jackson Laboratory [Stock No. 034860; B6.Cg-Tg(K18-ACE2)2Prlmn/J; genetic background (C57BL/6J x SJL/J)F2]. Immunogenicity experiments were performed in the animal facility of the Centro Nacional de Biotecnología (CNB-CSIC, Madrid, Spain), and efficacy experiments were conducted in the biosafety level 3 (BSL-3) facilities of the Centro de Investigación en Sanidad Animal (CISA)-Instituto Nacional de Investigaciones Agrarias (INIA)-Consejo Superior de Investigaciones Científicas (CSIC), Valdeolmos, Madrid, Spain. The animal studies were approved by the Ethical Committees of Animal Experimentation (CEEA) of the CNB-CSIC, and CISA-INIA-CSIC, and by the Division of Animal Protection of the Comunidad de Madrid (PROEX 49/20, 169.4/20, and 161.5/20). Animal procedures were conducted in accordance with international guidelines, local legislation, institutional requirements, and Spanish law under the Royal Decree (RD) 53/2013.

### Cells

DF-1 cells (a spontaneously immortalized chicken embryo fibroblast [CEF] cell line; ATCC CRL-12203) and HeLa cells (human epithelial cervix adenocarcinoma; ATCC CCL-2) were cultured in Dulbecco’s modified Eagle’s medium (DMEM) (Gibco-Life Technologies) supplemented with penicillin (100 U/mL; Sigma-Aldrich), streptomycin (100 mg/mL; Sigma-Aldrich), L-glutamine (2 mM; Sigma-Aldrich), non-essential amino acids (0.1 mM; Sigma-Aldrich), and 10% heat-inactivated fetal bovine serum (FBS) (Gibco-Life Technologies). Vero-E6 cells (from African green monkey kidney, ATCC CRL-1586) were maintained in DMEM supplemented with HEPES (10 mM; Gibco-Life Technologies), non-essential amino acids (0.1 mM; Sigma-Aldrich), penicillin (100 U/mL; Sigma-Aldrich), streptomycin (100 mg/mL; Sigma-Aldrich), and 10% heat inactivated FBS. Vero/TMPRSS2 cells (Vero-E6 cells constitutively expressing TMPRSS2 serine protease under geneticin selection, in order to be highly susceptible to SARS-CoV-2 infection) were maintained in DMEM supplemented with HEPES (10 mM; Gibco-Life Technologies), non-essential amino acids (0.1 mM; Sigma-Aldrich), penicillin (100 U/mL; Sigma-Aldrich), streptomycin (100 mg/mL; Sigma-Aldrich), Geneticin (G418, 1 mg/mL; Merck-Life Sciences), and 10% heat inactivated FBS. All cell cultures were maintained at 37 °C in a humidified incubator with 5% CO_2_.

### Viruses

We used the attenuated MVA wild-type (MVA-WT) poxvirus strain, derived from the Chorioallantois vaccinia virus Ankara strain (25), as the parental virus to generate the MVA-S(3P_XBB.1.5_) vaccine candidate. This recombinant expresses a human codon-optimized, full-length prefusion-stabilized SARS-CoV-2 S protein derived from the Omicron XBB.1.5 variant, containing 3 mutations in the furin cleavage site (R682G, R683S, and R685S) to prevent cleavage of the S protein in S1 and S2 domains, and 3 proline (3P) substitutions in the S2 region (A942P, K986P, and V987P) to stabilize the S protein in a prefusion conformation. Moreover, we used MVA-S(3P) (9), expressing a human codon-optimized full-length prefusion-stabilized SARS-CoV-2 S protein from the Wuhan strain with the same furin cleavage site mutations and 3P substitutions as a control for virus characterization. All MVA viruses were amplified in permissive DF-1 cells to generate master virus seed stocks (passage 2 [P2]) and titrated in DF-1 cells by plaque immunostaining assay, as previously described (26). For animal inoculations, MVA viruses were expanded in DF-1 cells and purified by centrifugation through two 36% (wt/vol) sucrose cushions in 10 mM Tris-HCl (pH 9). All viral stocks were tested and confirmed to be free of contamination with mycoplasma (checked by Mycoplasma Gel Detection kit; Biotools), bacteria (checked by growth on LB plates without ampicillin), and fungi (checked by growth on Columbia blood agar plates; Oxoid).

The SARS-CoV-2 MAD6 isolate, similar to the Wuhan strain but containing the D614G mutation in the S protein (27), was kindly provided by Dr. José M. Honrubia and Prof. Luis Enjuanes (CNB-CSIC, Madrid, Spain). The SARS-CoV-2 beta (B.1.351) variant (hCoV-19/France/PDL-IPP01065i/2021) was obtained through the European Virus Archive-Global (EVAg) platform, a project funded by the European Union’s Horizon 2020 research and innovation programme under grant agreement No. 653316. The SARS-CoV-2 B.1.1.529 (BA.1) Omicron variant (hCoV-19/Belgium/rega-20174/2021, EPI_ISL_6794907) was provided by Prof. Piet Maes from KU Leuven (Belgium) through Dr. Robbert Boudewijns and Dr. Kai Dallmeier (KU Leuven, Belgium). The SARS-CoV-2 Omicron BA.5 (EPI_ISL_13424827) and XBB.1.5 (EPI_ISL_16939528) subvariants were provided by Prof. Rafael Delgado (Hospital Universitario 12 de Octubre, Madrid, Spain). SARS-CoV-2 stocks were amplified in Vero/TMPRSS2 cells at a multiplicity of infection (MOI) of 0.001 plaque-forming units (PFUs)/cell (passage 2). Supernatants were harvested at 72 h post-infection (hpi), cleared by centrifugation, aliquoted, and stored at -80 °C. Virus infectious titers were determined by a standard plaque assay or median tissue culture infectious dose (TCID_50_) assay in Vero-E6 cells, as previously described (9).

### Construction of plasmid transfer vector pCyA-S(3P_XBB.1.5_) and generation of MVA-S(3P_XBB.1.5_) vaccine candidate

The plasmid transfer vector pCyA-S(3P_XBB.1.5_) was designed to generate the recombinant MVA-S(3P_XBB.1.5_) vaccine candidate. A human codon-optimized DNA sequence encoding the full-length prefusion-stabilized SARS-CoV-2 Omicron XBB.1.5 S protein, containing 3 mutations in the furin cleavage site (R682G, R683S, and R685S) and 3P substitutions in the S2 region (A942P, K986P, and V987P), was inserted into the thymidine kinase (TK) locus of parental MVA-WT under the control of the viral synthetic early/late (sE/L) promoter and preceded by a Kozak sequence (GCCACC). Briefly, a 3,813-bp DNA fragment encoding the SARS-CoV-2 Omicron XBB.1.5 full-length prefusion-stabilized S gene was synthesized by GeneArt (Thermo Fisher Scientific) and cloned into the pCyA plasmid vector (28), generating pCyA-S(3P_XBB.1.5_) (11,313 bp). This plasmid contains a β-galactosidase (β-Gal) reporter gene between two repeats of the left TK-flanking arm, allowing marker deletion by homologous recombination after serial passages.

DF-1 cells (3 × 10^6^ cells) were infected with parental MVA-WT virus at an MOI of 0.01 PFUs/cell and transfected 1 h later with 10 μg of pCyA-S(3P_XBB.1.5_) using Lipofectamine 2000 (Invitrogen), according to the manufacturer’s recommendations. At 72 hpi, cells were harvested, lysed by freeze-thaw cycles, sonicated, and used for recombinant virus screening. Recombinant MVA-S(3P_XBB.1.5_) viruses containing the SARS-CoV-2 Omicron XBB.1.5 full-length prefusion-stabilized S gene, inserted in the TK locus, and transiently coexpressing the β-Gal marker gene were selected by three consecutive rounds of plaque purification in DF-1 cells stained with X-Gal (5-bromo-4-chloro-3-indolyl-β-D-galactopyranoside, 1.2 mg/mL) (Sigma-Aldrich). In subsequent plaque purification rounds, recombinant MVA-S(3P_XBB.1.5_) viruses with the β-Gal gene deleted by homologous recombination between the left TK arm and the short-left TK arm repeat flanking the marker were isolated by three additional consecutive rounds of plaque purification screening for non-staining viral foci in DF-1 cells in the presence of X-Gal (1.2 mg/mL). Plaques isolated at each round were amplified in DF-1 cells, and the resulting virus stocks were used for the next purification round. Final recombinant MVA-S(3P_XBB.1.5_) virus stocks were grown, purified, and titrated as described above.

### Expression of SARS-CoV-2 S protein by western blotting

To evaluate SARS-CoV-2 S protein expression by MVA-S(3P_XBB.1.5_), monolayers of human HeLa cells grown in 24-well plates were infected at 5 PFUs/cell with MVA-S(3P_XBB.1.5_), MVA-S(3P), or control MVA-WT in the presence or absence of tunicamycin (10 μg/mL; Sigma-Aldrich), an inhibitor of N-glycosylation. At 6 or 24 hpi, equal amounts of cell extracts were lysed under reducing (1X Laemmli plus β-mercaptoethanol; Sigma-Aldrich) or nonreducing conditions (1X Laemmli without β-mercaptoethanol; Sigma-Aldrich), fractionated by 8% sodium dodecyl sulfate-polyacrylamide gel electrophoresis (SDS-PAGE), and analysed by Western blot. Membranes were probed with a rabbit polyclonal anti-SARS-CoV-2 receptor binding domain (RBD) antibody (Genetex; 1:1,000) to detect S protein expression. A rabbit anti-vaccinia virus polyclonal antibody (Invitrogen, 1:1,000) was used as a viral loading control. An anti-rabbit horseradish peroxidase (HRP)-conjugated antibody (Sigma-Aldrich; 1:5,000) was used as the secondary antibody. Stability of S protein expression by MVA-S(3P_XBB.1.5_) was also analysed by Western blot after 9 consecutive passages in DF-1 cells, as previously reported (9).

### Analysis of virus growth

To study the virus growth profile of MVA-S(3P_XBB.1.5_) in comparison to that of MVA-S(3P) and MVA-WT, monolayers of permissive DF-1 cells were infected at 0.01 PFUs/cell with MVA-S(3P_XBB.1.5_), MVA-S(3P), or MVA-WT, harvested at different times post-infection (0, 24, 48, and 72 hpi), and viral titers in cell lysates were determined by a plaque immunostaining assay, as described above.

### Mouse immunization, challenge, and sample collection

Groups of female K18-hACE2 mice (n = 15/group; 9 weeks old at study initiation) were immunized intramuscularly in both hind legs with either 0.5 µg of Comirnaty^®^ Omicron XBB.1.5 mRNA vaccine (Pfizer-BioNTech) or 1 × 10^7^ PFUs of MVA-S(3P_XBB.1.5_) in 100 µL PBS (50 µL per leg). The 0.5 µg mRNA and 1 × 10^7^ PFU MVA vaccine doses were selected based on previous preclinical studies demonstrating robust immunogenicity of these respective doses in mice (9, 12, 29–31). These doses were not intended to represent clinically dose-matched or proportionally equivalent formulations. Four weeks after priming (day 28), mice received the corresponding homologous or heterologous booster dose. Vaccination regimens included homologous mRNA_XBB.1.5_/mRNA_XBB.1.5_, homologous MVA-S(3P_XBB.1.5_)/MVA-S(3P_XBB.1.5_), heterologous mRNA_XBB.1.5_/MVA-S(3P_XBB.1.5_), or a single-dose MVA-S(3P_XBB.1.5_) regimen. PBS-inoculated mice served as controls. No adverse effects were observed in immunized animals throughout the study.

To assess cellular immune responses, 5 mice per group were euthanized on days 38 (10 days after the last vaccination) and 308 (9 months after the last vaccination) by CO_2_ inhalation. Spleens were collected, mechanically dissociated, depleted of red blood cells, and filtered through 40-µm cell strainers to obtain single-cell suspensions.

To evaluate SARS-CoV-2-specific humoral immune responses, blood was collected from each mouse on days 38 and 308 by cardiac puncture, and on days 14, 70, 98, 161, and 245 by submandibular bleeding. Blood samples were incubated at 37 °C for 1 h, kept overnight at 4 °C, and centrifuged at 3,600 rpm for 20 min at 4 °C to obtain serum. Serum samples were heat-inactivated at 56 °C for 30 min and stored at -20 °C until use.

On day 308 (9 months after the last vaccination), 5 mice per group were anesthetized with inhaled isoflurane (2–3% in oxygen) and challenged intranasally with 1 × 10^5^ PFUs of the SARS-CoV-2 Omicron XBB.1.5 variant in 50 µL PBS. Mice were monitored daily for body weight, clinical signs, and mortality for 3 days. At day 3 post-challenge (day 311), mice were euthanized by CO_2_ asphyxiation using a chamber fill rate of 20–30% of the chamber volume per minute, followed by cervical dislocation as a secondary physical method to confirm death, in accordance with institutional and national animal welfare guidelines. Following euthanasia, lung tissue, nasal washes (NW), and serum samples were collected. The entire left lung lobe was fixed in zinc formalin (Sigma-Aldrich) for 48 h and embedded in paraffin for histopathology. Right lung lobes were bisected longitudinally: one half was stored in RNAlater^®^ (Sigma-Aldrich) at -80 °C for RNA extraction, and the other half was weighed and stored at -80 °C for virus titration. NW samples were collected by flushing 400 µL PBS through the nasal cavity and stored at -80 °C until infectious virus quantification.

### Enzyme-linked immunosorbent assay

The titers of SARS-CoV-2 S–specific IgG antibodies in individual or pooled sera from immunized mice were determined by ELISA, as previously described (9). Endpoint IgG titers were defined as the highest serum dilution yielding an absorbance at 450 nm at least three times higher than that of naive serum. The recombinant soluble SARS-CoV-2 S proteins used to coat the plates derived from the Wuhan strain (GenBank accession number MN908947.3), the beta (B.1.351) variant (GISAID: EPI_ISL_712096), and the Omicron BA.1 (OL672836.1), BA.5 (ON249995.1), and XBB.1.5 (OP790748.1) subvariants. Expression plasmids encoding these soluble S proteins were generated, and proteins were expressed in mammalian cells and purified from culture supernatants as previously described (12).

### Neutralization of live SARS-CoV-2 virus

The capacity of individual or pooled sera obtained from immunized mice to neutralize live SARS-CoV-2 virus was evaluated using a microneutralization test (MNT) assay performed in a BSL-3 laboratory at the CNB-CSIC, as previously described (9). Briefly, serial dilutions of serum samples in DMEM supplemented with 2% FBS were incubated at a 1:1 ratio with 100 TCID_50_ of the indicated SARS-CoV-2 variants in 96-well plates for 1 h at 37 °C. Then, serum-virus mixtures were added in triplicate to Vero-E6 cell monolayers seeded in 96-well plates at 1.2 x 10^4^ cells per well. After 72 h of incubation, cells were fixed and stained with 5% crystal violet (Sigma-Aldrich). Plates were subsequently dried, and cell monolayers were solubilized with 1% SDS (Sigma-Aldrich). Absorbance was measured at 570 nm to determine the percentage of neutralization. The 50% neutralization titers (NT_50_), along with half maximal effective concentration (EC_50_) values and 95% confidence intervals (CI), were calculated using a nonlinear regression model fit with settings for agonist concentration versus normalized response curve using GraphPad Prism version 10.6.1.

### Antibody-dependent cellular phagocytosis

Antibody-dependent cellular phagocytosis (ADCP) activity was evaluated using individual serum samples from immunized mice. SARS-CoV-2 S proteins from the Omicron XBB.1.5 variant was covalently coupled to fluorescent beads (1 µm Red Carboxylate-Modified FluoSpheres; Invitrogen) according to the manufacturer’s instructions. Bead-antigen complexes were incubated with serum samples diluted 1:10 in PBS for 2 h at 37 °C to allow immune complex formation, followed by washing with PBS. THP-1 cells were then added at 1 × 10^5^ cells/mL and incubated overnight at 37 °C with 5% CO_2_. Phagocytic uptake of immune complex-coated beads by THP-1 cells was quantified by flow cytometry using a CytoFLEX cytometer (Beckman Coulter Life Sciences). All samples were analyzed in triplicate.

### Antibody-dependent complement deposition

Antibody-dependent complement deposition (ADCD) was assessed in individual serum samples from immunized mice, using fluorescent bead-based assays. Red Carboxylate-Modified FluoSpheres (1 µm; Invitrogen) were covalently coupled to SARS-CoV-2 S proteins from the Wuhan strain or the Omicron XBB.1.5 variant. Immune complexes were generated as described for ADCP. After washing, lyophilized guinea pig complement (Sigma-Aldrich), diluted 1:25 in RPMI supplemented with 10% FBS, was added and samples were incubated for 20 min at 37 °C. Complement C3 deposition on bead surfaces was detected by staining with FITC-conjugated anti-guinea pig C3 antibody (Invitrogen) and quantified by flow cytometry using a CytoFLEX cytometer (Beckman Coulter Life Sciences).

### Antibody-dependent natural killer cell activation

Antibody-dependent natural killer (NK) cell activation (ADNKA) was evaluated using individual serum samples from immunized mice, as previously described (32). Briefly, 96-well NUNC MaxiSorp plates (Thermo Scientific) were coated overnight at 4°C with SARS-CoV-2 S protein (Wuhan or Omicron XBB.1.5) at 3 μg/mL in borate-buffered saline (BBS). Plates were washed 3 times with PBS-Tween and blocked overnight at 4 °C with PBS containing 1% casein (PBS-C). Serum samples diluted in PBS-C were then added and incubated for 2 h at room temperature. Plates were then washed with PBS-Tween and PBS, and mouse NK cells isolated from mouse splenocytes using a negative selection kit (NK Cell Isolation Kit; Miltenyi Biotec) were added at 5 × 10^4^ cells per well in RPMI supplemented with 10% FBS, monensin (1×), and anti-CD107a-FITC (1 µg/mL). NK cells were previously expanded for 7 days in complete RPMI supplemented with 1% non-essential amino acids (Gibco), 1 mM MEM sodium pyruvate (Gibco), 50 µM β-mercaptoethanol, 1000 U/mL mouse IL-2, and 50 ng/mL mouse IL-15 at 37°C and 5% CO_2_, with restimulation on days 3 and 5 with IL-2 and IL-15. After 5 h incubation at 37°C and 5% CO_2_, cells were stained with anti-CD3-PE-CF594 and NK1.1-PE antibodies in PBA for 30 min, washed with PBS, fixed/permeabilized with BD Cytofix/Cytoperm™ Fixation/Permeabilization Kit, and stained intracellularly with anti-IFN-γ-PE-Cy7 and anti-TNF-α-APC antibodies. Samples were acquired on a CytoFLEX cytometer (Beckman Coulter Life Sciences).

### Intracellular cytokine staining assay

The magnitude, polyfunctionality, and memory phenotype of SARS-CoV-2 S-specific CD4^+^ and CD8^+^ T cells expressing CD107a and/or producing IFNγ, TNFα, and/or IL-2 were analyzed by an ICS assay, as previously described (12). Splenocytes were stimulated with two peptide pools spanning the full-length SARS-CoV-2 Omicron XBB.1.5 S protein (1 µg/mL; JPT Peptide Technologies), consisting of consecutive 15-mer peptides overlapping by 11 amino acids and covering the S1 and S2 regions. Stimulation was performed in the presence of brefeldin A (BD Biosciences), monensin (BD Biosciences) and anti-CD107a-FITC (1µg/mL). Cells were subsequently stained for surface markers (CD3, CD4, CD8, CD127, and CD62L), followed by fixation and permeabilization, and intracellular staining for cytokines (IFN-γ, IL-2, and TNF-α), Samples were acquired on a CytoFLEX cytometer (Beckman Coulter Life Sciences), and data were analyzed using FlowJo software (version 10.4.2; Tree Star).

### Analysis of viral yields by plaque assay

Lung and NW samples from K18-hACE2 mice collected at day 3 post-challenge were analyzed for the presence of infectious SARS-CoV-2 by plaque assay, as previously described (9). Lungs were harvested, weighed, and stored at -80 °C until homogenization with a gentleMACS dissociator (Miltenyi Biotec) in 2 mL PBS. Undiluted and serial ten-fold dilutions of lung homogenates or NW samples were added in triplicate to Vero-E6 cell monolayers seeded in 12-well plates at 5 x 10^5^ cells/well. After 1 h adsorption, the inoculum was removed, and cells were overlaid with a mixture of DMEM 2X-4% FBS and Avicel^®^ RC-591 (2:1; DuPont Nutrition Biosciences ApS). Plates were incubated at 37 °C with 5% CO_2_ for 3 days. Cells were then fixed with 10% formaldehyde (Sigma-Aldrich) for 1 h, the supernatant was removed, and plaques were visualized by staining with 0.5% crystal violet (Sigma-Aldrich). SARS-CoV-2 titers were expressed as PFUs per gram of lung tissue or per mL of NW.

### Quantification of SARS-CoV-2 RNA by reverse transcription-quantitative polymerase chain reaction

Total RNA was extracted from lung samples from K18-hACE2 mice collected at day 3 post-challenge. Lung tissues stored in RNAlater (Sigma-Aldrich) at -80 °C were homogenized using a gentleMACS dissociator (Miltenyi Biotec) in 2 mL of RLT buffer (Qiagen) supplemented with β-mercaptoethanol (Sigma-Aldrich), as previously described (9). Total RNA was isolated 600 μL of homogenized lung tissue using the RNeasy Mini Kit (Qiagen), according to the manufacturer’s specifications. SARS-CoV-2 RNA levels were quantified by RT-qPCR, following a previously described method (9), using validated primer/probe sets targeting subgenomic RNA encoding the E protein (33) and a conserved region of ORF1 (34). Gene expression was normalized to the cellular 28S ribosomal RNA gene. Relative viral RNA levels were calculated using the 2^−ΔΔCt^ method, normalized to uninfected controls. All samples were analyzed in duplicate.

### Transcriptome analysis

RNA concentration and integrity were assessed using the Qsep100 system (BiOptic), and samples with RNA integrity number (RIN) < 7 were excluded. Library preparation, short-red RNA sequencing, and quality control were performed by BGI (BGI Genomics Co., Ltd. Shenzhen, Guangdong, China), generating approximately 30 million reads per sample using the DNBSEQ™ next-generation sequencing platform.

Raw sequencing data were filtered using fastp v1.0.1 to remove adapter sequences and low-quality reads (35), retaining reads with a Phred quality score > 30 and a minimum length of 36 bp. Filtered reads were aligned to the *Mus musculus* reference genome (genome assembly GRCm39) using subread-align, and gene-level read counts were obtained with featureCounts, both implemented in the Subread v2.1.1 package (36). Genes with low expression levels (fewer than 15 reads per group) were excluded. Normalization and differential expression (DE) analysis were performed using edgeR v4.4.0 (37). Each vaccinated group was compared with the PBS/PBS infection control group. An additional non-infected control group was also included for comparison. Multiple testing correction was applied using the false discovery rate (FDR) approach, and genes with a q-value < 0.05 were considered differentially expressed. The complete list of DEGs identified in each comparison, including normalized expression values, log2 fold changes, and statistical test results, is provided in **Supplementary Table 1**.

### Statistical procedures

All graphs and statistical analyses were performed using GraphPad Prism version 10.6.1. Ordinary two-way ANOVA followed by Tukey’s multiple comparisons test on log-transformed data was used to analyze anti-S XBB.1.5 IgG titers and NT_50_ titers from individual serum samples, as well as viral RNA levels in lung tissues. Ordinary two-way ANOVA followed by Tukey’s multiple comparisons test was used for comparisons of ICS-derived S-specific CD4^+^ and CD8^+^ T-cell responses. ADCP, ADCD, and ADNKA data were analyzed using ordinary one-way ANOVA followed by Tukey’s multiple comparisons test. Viral titers in lung tissues and NWs were analyzed using log-transformed data with ordinary one-way ANOVA followed by Tukey’s multiple comparisons test. Data generated from pooled serum samples were analyzed descriptively, and no inferential statistical analyses were performed because each vaccination group was represented by a single pooled biological sample. Statistical significance was defined as follows: *p < 0.033; **p < 0.002; ***p < 0.0002; ****p < 0.0001.

## Results

### Generation of an MVA-based vaccine candidate expressing a prefusion-stabilized SARS-CoV-2 Omicron XBB.1.5 S protein

**Figure 1:**
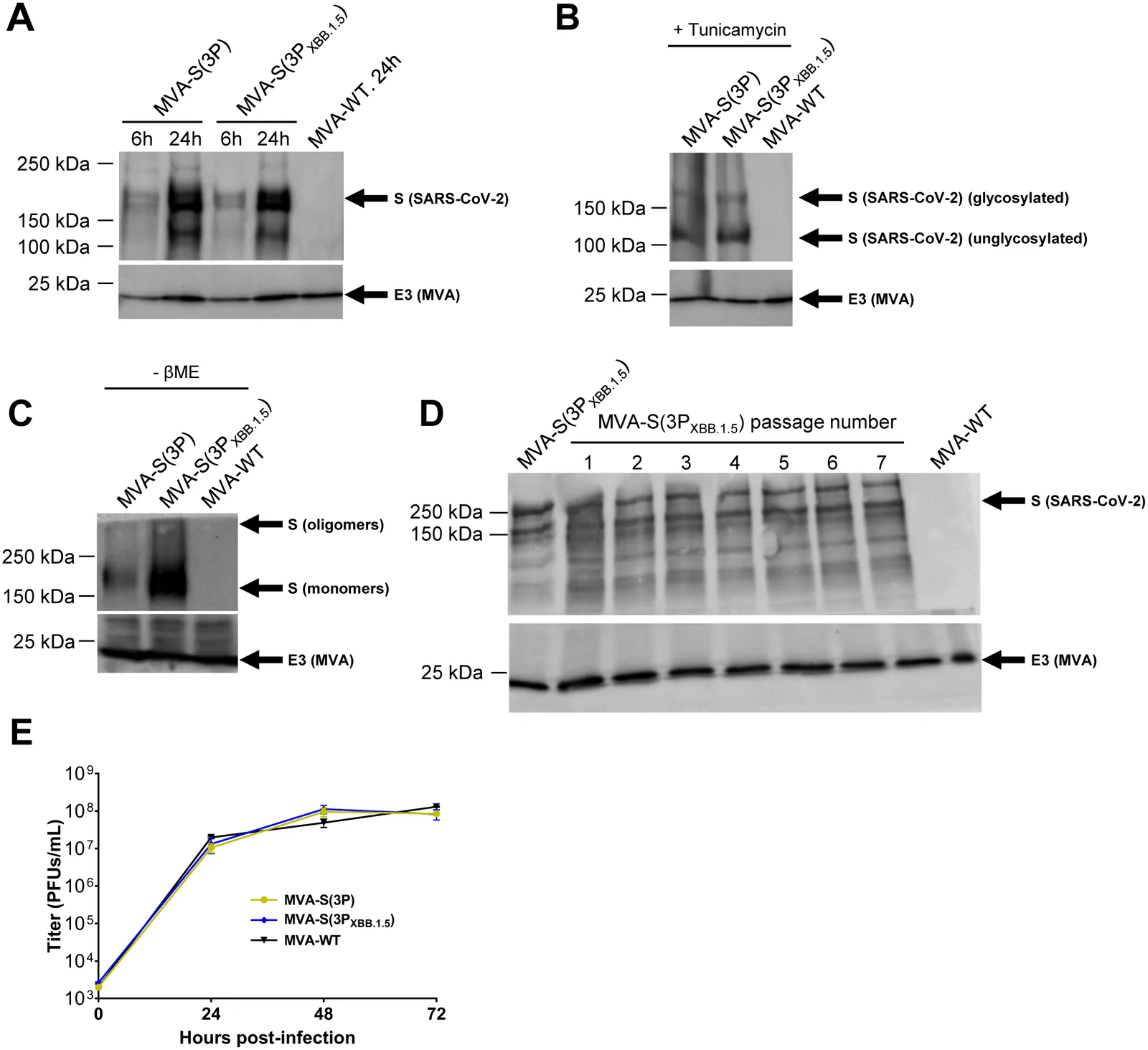
Design, generation, and *in vitro* characterization of the MVA-S(3P_XBB.1.5_) vaccine candidate. **(A-C)** Expression of SARS-CoV-2 S protein by the MVA-S(3P_XBB.1.5_) vaccine candidate in human HeLa cells. Rabbit polyclonal anti-RBD and anti-MVA E3 antibodies were used for protein detection by Western blot following SDS-PAGE. Molecular weight markers (kDa) are indicated. **(A)** Western blot analysis of MVA-infected HeLa cell extracts (5 PFUs/cell) at 6 and 24 h post-infection, under reducing conditions (plus β-mercaptoethanol). **(B)** Western blot analysis of MVA-infected HeLa cell extracts (5 PFUs/cell) at 24 h post-infection, treated with tunicamycin and analysed under reducing conditions (plus β-mercaptoethanol). Detection of glycosylated and unglycosylated S proteins is indicated. **(C)** Western blot analysis of MVA-infected HeLa cell extracts (5 PFUs/cell) at 24 h post-infection, under non-reducing conditions (without β-mercaptoethanol). Detection of S protein oligomers and monomers is indicated. **(D)** Genetic stability of the MVA-S(3P_XBB.1.5_) vaccine candidate. Western blot analysis of DF-1 cell extracts (24 h post-infection) infected with the master virus seed stock (P2) of MVA-S(3P_XBB.1.5_) and after seven serial passages at low MOI in DF-1 cells. Samples were analysed under reducing conditions (plus β-mercaptoethanol). **(E)** Viral growth kinetics of MVA-S(3P_XBB.1.5_). DF-1 cell monolayers were infected at 0.01 PFUs/cell with MVA-WT, MVA-S(3P), and MVA-S(3P_XBB.1.5_). At the indicated times post-infection (0, 24, 48, and 72 h), virus titers (PFUs/mL) in cell lysates were determined by plaque immunostaining assay. Data represent the mean of two independent experiments.

We generated a novel MVA-based vaccine candidate targeting the Omicron XBB.1.5 variant, termed MVA-S(3P_XBB.1.5_), expressing a full-length prefusion-stabilized Omicron XBB.1.5 S protein containing three proline (3P) substitutions in the S2 region (A942P, K986P, and V987P) and mutations in the furin cleavage site (R682G, R683S, and R685S). The expression profile of the S protein was examined at different time points in non-permissive human HeLa cells infected with MVA-S(3P_XBB.1.5_) or MVA-S(3P), which expresses a prefusion-stabilized S protein from the Wuhan strain. Western blot analysis revealed a major ~180 kDa protein corresponding to the glycosylated S monomer (**Figure 1A**). Treatment with tunicamycin reduced its molecular weight, confirming glycosylation (**Figure 1B**). Furthermore, under non-reducing conditions, the S protein displayed higher molecular-weight species consistent with the formation of oligomers, presumably trimers (**Figure 1C**). Next, to assess expression stability within the MVA vector, SARS-CoV-2 S protein levels were analyzed over 7 consecutive cell passages in permissive chicken DF-1 cells infected with MVA-S(3P_XBB.1.5_) at low multiplicity of infection (MOI) (0.05 PFUs/cell), demonstrating sustained S protein expression throughout serial passaging (**Figure 1D**).

Finally, we compared the growth kinetics of MVA-S(3P_XBB.1.5_), MVA-S(3P), and MVA-WT in DF-1 cells. All viruses exhibited similar replication profiles, indicating that constitutive expression of the S protein does not impair MVA replication under permissive conditions (**Figure 1E**).

### Homologous and heterologous mRNA/MVA vaccination against SARS-CoV-2 XBB.1.5 elicits robust, broad, and durable S-specific IgG responses

**Figure 2:**
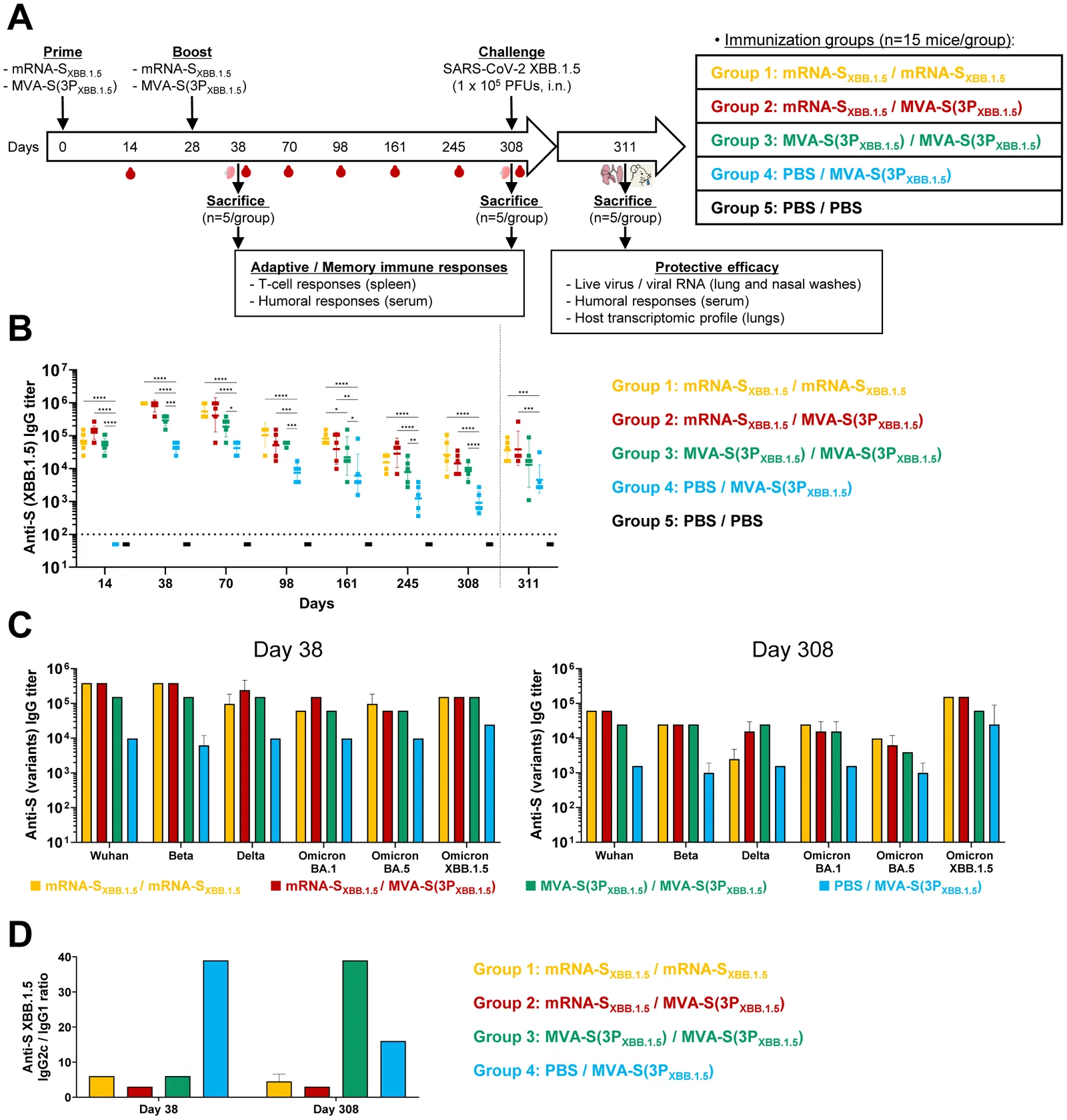
Immunization strategy and longitudinal SARS-CoV-2 S-specific IgG responses induced by homologous and heterologous mRNA/MVA vaccination. **(A)** Experimental design. Female K18-hACE2 transgenic mice (n=15 per group) were primed intramuscularly with mRNA-S_XBB.1.5_ (Comirnaty^®^ Omicron XBB.1.5) or MVA-S(3P_XBB.1.5_), followed by homologous or heterologous boosting at week 4 (day 28). Immunization groups are indicated: mRNA-S_XBB.1.5_/mRNA-S_XBB.1.5_, mRNA-S_XBB.1.5_/MVA-S(3P_XBB.1.5_), MVA-S(3P_XBB.1.5_)/MVA-S(3P_XBB.1.5_), and single-dose MVA-S(3P_XBB.1.5_) (PBS/MVA-S(3P_XBB.1.5_), with PBS/PBS as infection control. Subsets of 5 mice per group were sacrificed on days 38 and 308 to assess adaptive and memory immune responses, respectively (T-cell responses in spleen and humoral responses in serum samples), and additional blood samples were collected by submandibular bleeding at multiple time points for humoral response analysis (days 14, 70, 98, 161, and 245). On day 308, another subset of animals (5 mice per group) was challenged intranasally with 1 x 10^5^ PFUs of SARS-CoV-2 Omicron XBB.1.5. At day 3 post-challenge (day 311), mice were sacrificed and lungs, nasal washes, and serum samples were collected. **(B)** Serum IgG titers against SARS-CoV-2 S XBB.1.5. Measured at multiple time points by ELISA in individual mouse serum samples. Geometric mean ± geometric standard deviation (SD) of endpoint titers from duplicates of each sample for each group is shown. The dotted line represents the limit of detection. Ordinary two-way ANOVA followed by Tukey’s multiple comparisons test on transformed data: *p < 0.033; **p < 0.002; ***p < 0.0002; ****p < 0.0001. **(C)** IgG titers against S from various SARS-CoV-2 variants. Measured by ELISA, in pooled sera from each group collected at days 38 (left panel) and 308 (right panel). Geometric mean ± geometric SD of technical duplicate measurements from each pooled serum sample are shown. The dotted line represents the limit of detection. **(D)** IgG2c/IgG1 isotype ratios against XBB.1.5 S protein. Measured by ELISA in duplicate, using pooled sera from each group at days 38 and 308. The mean ± SD of IgG2c/IgG1 endpoint titer ratios for each group is shown.

Female K18-hACE2 transgenic mice (n=15/group) were primed intramuscularly with either Comirnaty^®^ Omicron XBB.1.5 (mRNA-S_XBB.1.5_; 0.5 µg/mouse) or MVA-S(3P_XBB.1.5_) (1 x 10^7^ PFUs/mouse). Two weeks later, mice were bled, and at week 4 received the corresponding homologous or heterologous immunizations (**Figure 2A**). Vaccination groups included mRNA-S_XBB.1.5_/mRNA-S_XBB.1.5_, mRNA-S_XBB.1.5_/MVA-S(3P_XBB.1.5_), MVA-S(3P _XBB.1.5_)/MVA-S(3P _XBB.1.5_), and a single-dose MVA-S(3P _XBB.1.5_) group, with PBS-inoculated mice as the control group. On day 38 (10 days after the last vaccination), mice (n=5/group) were sacrificed, and serum samples, draining lymph nodes, and spleens were collected to evaluate SARS-CoV-2 Omicron XBB.1.5 S-specific humoral and cellular immune responses, respectively. Additional animals (n=5/group) were bled on days 70, 98, 161, and 245 and sacrificed on day 308, when serum samples and spleens were similarly collected. The remaining animals (n=5/group) were challenged intranasally with 1 x 10^5^ PFUs of SARS-CoV-2 Omicron XBB.1.5 variant at day 308, corresponding to approximately 9 months after the last vaccination (**Figure 2A**).

To evaluate longitudinally the magnitude and durability of humoral responses elicited by the different vaccination strategies, SARS-CoV-2 S-specific serum IgG antibody titers were determined by ELISA at multiple time points (**Figure 2B**). Following the first dose (day 14), both mRNA-S_XBB.1.5_- and MVA-S(3P_XBB.1.5_)-immunized mice developed high (~10^5^) and comparable levels of anti-S IgG antibodies (**Figure 2B**). Ten days after the booster (day 38), all groups receiving two doses showed an approximately one-log increase in anti-S IgG titers, which were similar across the two-dose regimens and significantly higher than those observed in the single-dose MVA-S(3P_XBB.1.5_) group (**Figure 2B**). Between days 38 and 70 (10 and 42 days after the last vaccination), titers remained at peak levels (~10^6^) in all groups. From those peak levels until the challenge (day 308, around 9 months after the last vaccination), titers gradually declined by ~22-fold (~95.5%) in the mRNA-S_XBB.1.5_/mRNA-S_XBB.1.5_ group and ~46-fold (~97.8%) in the mRNA-S_XBB.1.5_/MVA-S(3P_XBB.1.5_) group, remaining comparable among all two-dose groups and significantly higher than in the single-dose MVA-S(3P_XBB.1.5_) group (**Figure 2B**). Following viral challenge (day 311), antibody levels increased minimally in the mRNA-S_XBB.1.5_/mRNA-S_XBB.1.5_ group (~1.05-fold), moderately in the mRNA-S_XBB.1.5_/MVA-S(3P_XBB.1.5_) and MVA-S(3P_XBB.1.5_)/MVA-S(3P_XBB.1.5_) groups (~4.6- and ~3.1-fold, respectively), and markedly in the single-dose MVA-S(3P_XBB.1._5) group (~7.6-fold). The limited increase in antibody titers upon challenge suggests that pre-existing immunity rapidly controlled viral replication, limiting antigen availability for further boosting of the humoral response.

Moreover, to assess the breadth of binding antibodies induced by the different immunization groups, IgG levels against S protein from various SARS-CoV-2 variants were measured by ELISA in pooled sera collected on days 38 and 308 (**Figure 2C**). At day 38, all vaccinated groups mounted broad humoral responses, generating high IgG titers against S protein from the Wuhan, Beta, Delta, Omicron BA.1, and BA.5 variants, as well as against the study variant, Omicron XBB.1.5, although with some differences among regimens and variants. All two-dose immunization groups elicited comparable titers against all Omicron subvariants tested, which were higher than those induced by the single-dose MVA-S(3P_XBB.1.5_) group (**Figure 2C**, left panel). In contrast, against more ancestral variants such as Wuhan and Beta, the mRNA-S_XBB.1.5_/mRNA-S_XBB.1.5_ and mRNA-S_XBB.1.5_/MVA-S(3P_XBB.1.5_) groups induced higher titers (**Figure 2C**, left panel). Notably, the mRNA-S_XBB.1.5_/MVA-S(3P_XBB.1.5_) regimen elicited the highest titers against the Delta variant (**Figure 2C**, left panel). By day 308, IgG titers declined in all vaccination groups across all variants. Titers against ancestral variants (Wuhan, Beta, and Delta) and earlier Omicron variants were slightly lower than those observed for Omicron XBB.1.5; however, similar relative titers were maintained across variants, indicating preserved breadth over time.

To further characterize the antibody response, anti-S Omicron XBB.1.5 IgG2c and IgG1 titers were measured in pooled sera from days 38 and 308 (10 days and 9 months after the last vaccination, respectively), and the corresponding IgG2c/IgG1 ratios were calculated as a surrogate marker of Th1/Th2 bias (**Figure 2D**). On day 38, the single dose MVA-S(3P_XBB.1.5_) group showed a higher anti-S Omicron XBB.1.5 IgG2c/IgG1 ratio (39:1). This was followed by the mRNA-S_XBB.1.5_/mRNA-S_XBB.1.5_ and MVA-S(3P_XBB.1.5_)/MVA-S(3P_XBB.1.5_) groups (both 6:1), and finally the mRNA-S_XBB.1.5_/MVA-S(3P_XBB.1.5_) group (3:1). By day 308, the IgG2c/IgG1 ratio in the mRNA-S_XBB.1.5_/MVA-S(3P_XBB.1.5_) group remained unchanged, whereas the ratio in the mRNA-S_XBB.1.5_/mRNA-S_XBB.1.5_ group shifted to 9:2 and that of the single-dose MVA-S(3P_XBB.1.5_) group decreased to 16:1. Notably, the MVA-S(3P_XBB.1.5_)/MVA-S(3P_XBB.1.5_) group showed a marked increase in the IgG2c/IgG1 ratio, reaching 39:1.

### Homologous and heterologous mRNA/MVA vaccination against SARS-CoV-2 XBB.1.5 induces potent, broad, and durable neutralizing antibody responses

**Figure 3:**
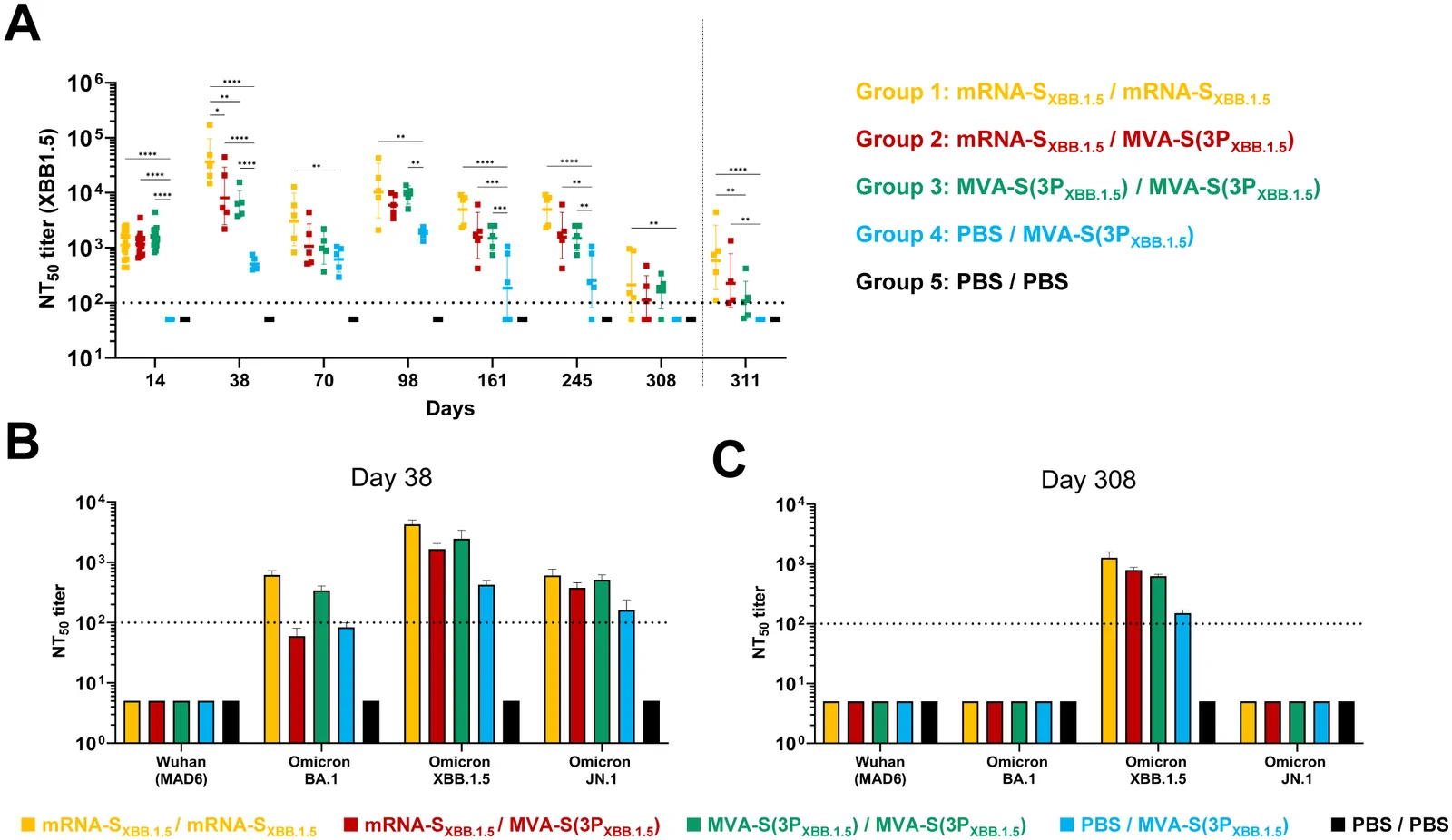
Homologous and heterologous mRNA/MVA vaccination induces broad and durable neutralizing antibody responses against SARS-CoV-2 XBB.1.5. **(A)** Longitudinal analysis of serum neutralizing activity measured by live-virus microneutralization (MNT) assay against SARS-CoV-2 XBB.1.5. NT_50_ titers were determined in triplicate, at the indicated time points in individual mice from each vaccination group. Geometric means ± SD of NT_50_ titers for each group are shown. The dotted line indicates the limit of detection. Ordinary two-way ANOVA followed by Tukey’s multiple comparisons test of transformed data: *p < 0.033; **p < 0.002; ***p < 0.0002; ****p<0.0001. **(B, C)** Cross-neutralization of SARS-CoV-2 MAD6 (Wuhan), Omicron BA.1, XBB.1.5, and JN.1. NT_50_ titers from pooled sera of each group collected at days 38 **(B)** and 308 **(C)** were analyzed in triplicate by live-virus MNT assay. Geometric means ± SD of NT_50_ titers for each pooled serum sample are shown. The dotted line represents the limit of detection.

To determine the functional quality of vaccine-induced antibodies over time, live-virus neutralization assays were performed using sera collected at multiple time points after the last vaccination (**Figure 3A**). After the first dose (day 14), both mRNA-S_XBB.1.5_- and MVA-S(3P_XBB.1.5_)-immunized mice developed high (~10^3^) and comparable neutralizing antibody titers (**Figure 3A**). On day 38 (10 days after the last vaccination), all groups exhibited increased neutralizing activity, although to different extents, reaching peak NT_50_ titers at this time point. The mRNA-S_XBB.1.5_/mRNA-S_XBB.1.5_ group showed the greatest increase in NT_50_ titers (~42.7-fold), which were significantly higher than those observed in the other two-dose groups; in turn, titers in these groups were significantly higher than those in the single-dose MVA-S(3P_XBB.1.5_) group (**Figure 3A**). Following this peak, NT_50_ titers gradually declined and became comparable among the three two-dose immunization groups from day 70 onward (**Figure 3A**). By day 308 (9 months after the last vaccination), titers had decreased by approximately two orders of magnitude, becoming undetectable in some samples (**Figure 3A**). In the group that received only one dose of MVA-S(3P_XBB.1.5_), NT_50_ titers fell below the limit of detection (LOD) in some mice from day 161 (nearly 5 months after the last vaccination) onward, and by day 308 all mice exhibited NT_50_ titers below the LOD (**Figure 3A**). Following viral challenge (day 311), NT_50_ titers in the three two-dose immunization groups showed a modest but highly variable increase among individual mice. At this time point, NT_50_ titers were significantly higher in the mRNA-S_XBB.1.5_/mRNA-S_XBB.1.5_ group than in the MVA-S(3P_XBB.1.5_)/MVA-S(3P_XBB.1.5_) group, whereas no significant differences were observed in the other comparisons among the two-dose groups (**Figure 3A**). In contrast, the group immunized with a single dose of MVA-S(3P_XBB.1.5_) did not exhibit any increase in NT_50_ titers after challenge (**Figure 3A**).

To further characterize the breadth of these neutralizing responses, NT_50_ titers were determined in pooled serum samples tested against different live SARS-CoV-2 variants on days 38 (10 days after the last vaccination) and 308 (9 months after the last vaccination) (**Figure 3B**). On day 38, all three two-dose immunization groups showed detectable neutralizing activity against Omicron JN.1, a later subvariant than XBB.1.5, although at slightly lower levels than those observed against the study variant (**Figure 3B**, left panel). Notably, only the mRNA-S_XBB.1.5_/mRNA-S_XBB.1.5_ and MVA-S(3P_XBB.1.5_)/MVA-S(3P_XBB.1.5_) groups showed detectable NT_50_ titers against Omicron BA.1; however, these levels were very low (<10³), and none of the groups displayed neutralizing antibodies against the ancestral Wuhan variant (**Figure 3B**, left panel). By day 308, no neutralizing antibodies were detected against any variant other than the study variant, Omicron XBB.1.5 (**Figure 3B**, right panel).

### Homologous and heterologous mRNA/MVA vaccination against SARS-CoV-2 XBB.1.5 triggers and sustains antibody-mediated effector functions

To complement virus neutralization analyses, antibody-mediated Fc effector functions were examined in serum samples obtained from vaccinated mice at early (day 38; 10 days after the last vaccination) and late (day 308; 9 months after the last vaccination) time points. These analyses included antibody-dependent cellular phagocytosis (ADCP), antibody-dependent complement deposition (ADCD), and antibody-dependent natural killer (NK) cell activation (ADNKA).

**Figure 4:**
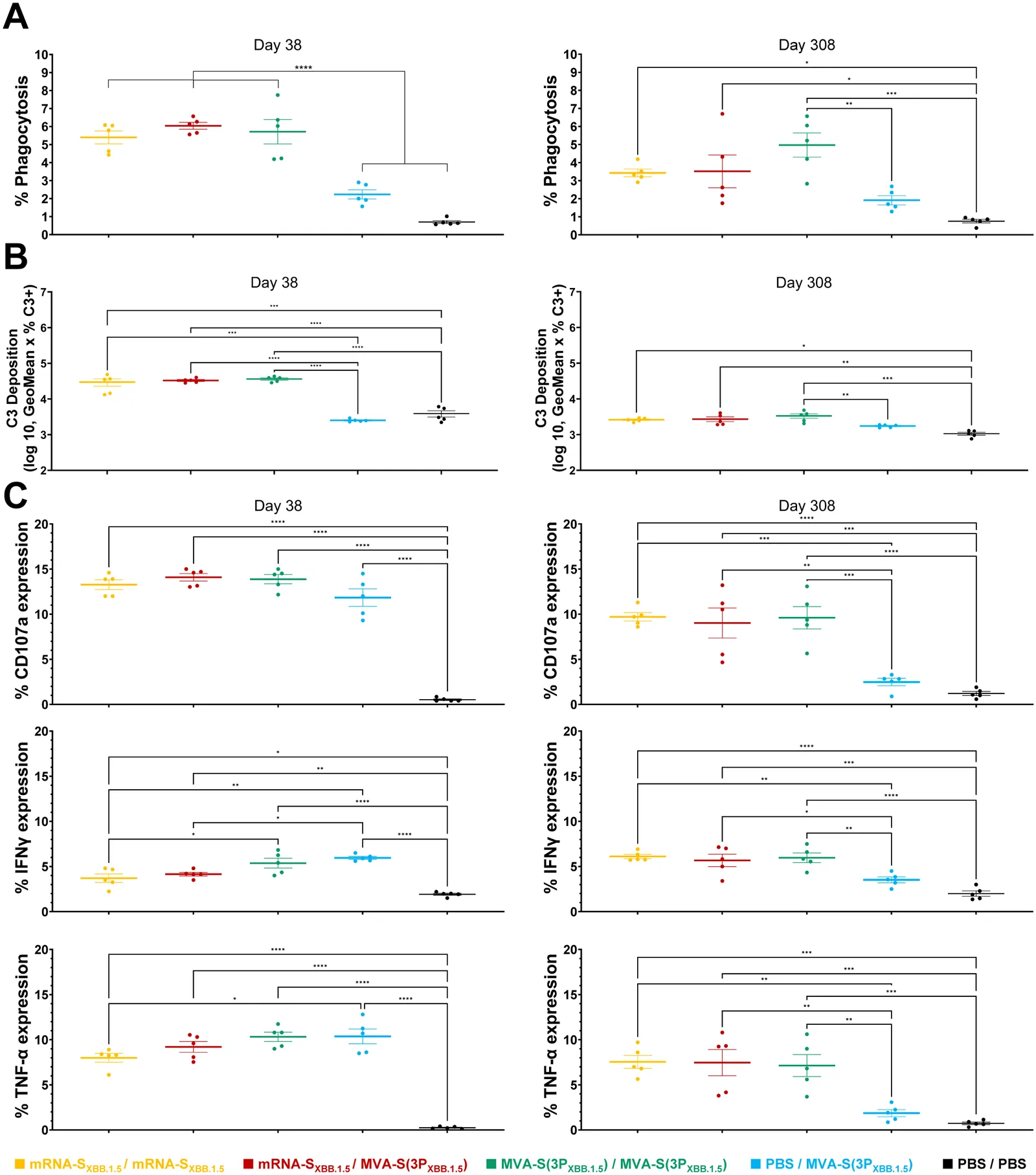
Homologous and heterologous mRNA/MVA vaccination induces sustained Fc-mediated antibody effector functions. **(A)** ADCP activity against fluorescent beads coated with SARS-CoV-2 S protein from Omicron XBB.1.5. Data from individual serum samples from each group collected on days 38 (left panel) and 308 (right panel). The mean ± SEM of phagocytosis percentages from triplicates is shown. Ordinary one-way ANOVA followed by Tukey’s multiple comparisons test: *p < 0.033; **p < 0.002; ***p < 0.0002; ****p<0.0001. **(B)** ADCD activity against fluorescent beads coated with SARS-CoV-2 S protein from Omicron XBB.1.5. Data from individual serum samples from each group collected on days 38 (left panel) and 308 (right panel). The mean ± SEM of C3 deposition from triplicates is shown. Ordinary one-way ANOVA followed by Tukey’s multiple comparisons test: *p < 0.033; **p < 0.002; ***p < 0.0002; ****p<0.0001. **(C)** ADNKA against SARS-CoV-2 S proteins from Omicron XBB.1.5. Data from individual serum samples from each group collected on days 38 (left panels) and 308 (right panels). The mean ± SEM of the percentage of NK cells expressing the CD107a degranulation marker (upper panels) or producing IFNγ (middle panels) or TNF-α (lower panels) is shown. Ordinary one-way ANOVA followed by Tukey’s multiple comparisons test: *p < 0.033; **p < 0.002; ***p < 0.0002; ****p<0.0001.

In the ADCP assay, at day 38, comparable phagocytic activity was observed among antibodies elicited by the three two-dose vaccination regimens, and was significantly higher than that induced by a single dose of MVA-S(3P_XBB.1.5_) (**Figure 4A**, left panel). Importantly, this activity showed only a modest decline over the 9-month study period, decreasing to approximately 3.5% in the mRNA-S_XBB.1.5_-primed groups and remaining close to 5% in the MVA-S(3P_XBB.1.5_)/MVA-S(3P_XBB.1.5_) group at day 308 (**Figure 4A**, right panel). Although differences among the two-dose regimens were not statistically significant, all remained significantly higher than those induced by a single dose of MVA-S(3P_XBB.1.5_), which exhibited phagocytic activity of around 2% at day 308, similar to levels observed at day 38.

In the ADCD assay, the three two-dose vaccination regimens induced comparable levels of C3 deposition at day 38, which were significantly higher than those induced by a single dose of MVA-S(3P_XBB.1.5_) (**Figure 4B**, left panel). By day 308, C3 deposition decreased to similar levels across all vaccinated groups, while remaining significantly higher than in the PBS/PBS control group (**Figure 4B**, right panel).

In the ADNKA assay, antibodies present in sera from all four immunization groups mediated, at day 38, comparable levels of the degranulation marker CD107a in NK cells, which were significantly higher than those observed in sera from non-immunized (PBS/PBS) mice (**Figure 4C**, left panels and **Supplementary Figure 1A**, left panels). Interestingly, at this time point, the MVA-S(3P_XBB.1.5_)/MVA-S(3P_XBB.1.5_) and PBS/MVA-S(3P_XBB.1.5_) groups exhibited higher expression of IFNγ and TNFα than the mRNA-S_XBB.1.5_/mRNA-S_XBB.1.5_ and mRNA-S_XBB.1.5_/MVA-S(3P_XBB.1.5_) groups, although the magnitude of both cytokine responses was markedly lower than that of CD107a (**Figure 4C**, left panels and **Supplementary Figure 1A**, left panels). However, by 9 months after the last vaccination, CD107a, IFNγ and TNFα expression in mice receiving a single dose of MVA-S(3P_XBB.1.5_) declined to levels similar to those of naïve mice, whereas levels in the two-dose immunization groups remained significantly elevated (**Figure 4C**, right panels and **Supplementary Figure 1B**, right panels).

### Homologous and heterologous mRNA/MVA vaccination against SARS-CoV-2 XBB.1.5 promotes durable S-specific T cell responses

To assess T cell-mediated immune responses elicited by the different vaccination strategies, an intracellular cytokine staining (ICS) assay was performed on splenocytes collected on day 38 (10 days after the last vaccination) and day 308 (9 months after the last vaccination). *Ex vivo* stimulation was carried out using peptide pools spanning the entire Omicron XBB.1.5 SARS-CoV-2 S protein. The expression of the degranulation marker CD107a and the production of IFN-γ, TNF-α, and IL-2 were evaluated in both CD4^+^ and CD8^+^ T cell populations.

**Figure 5:**
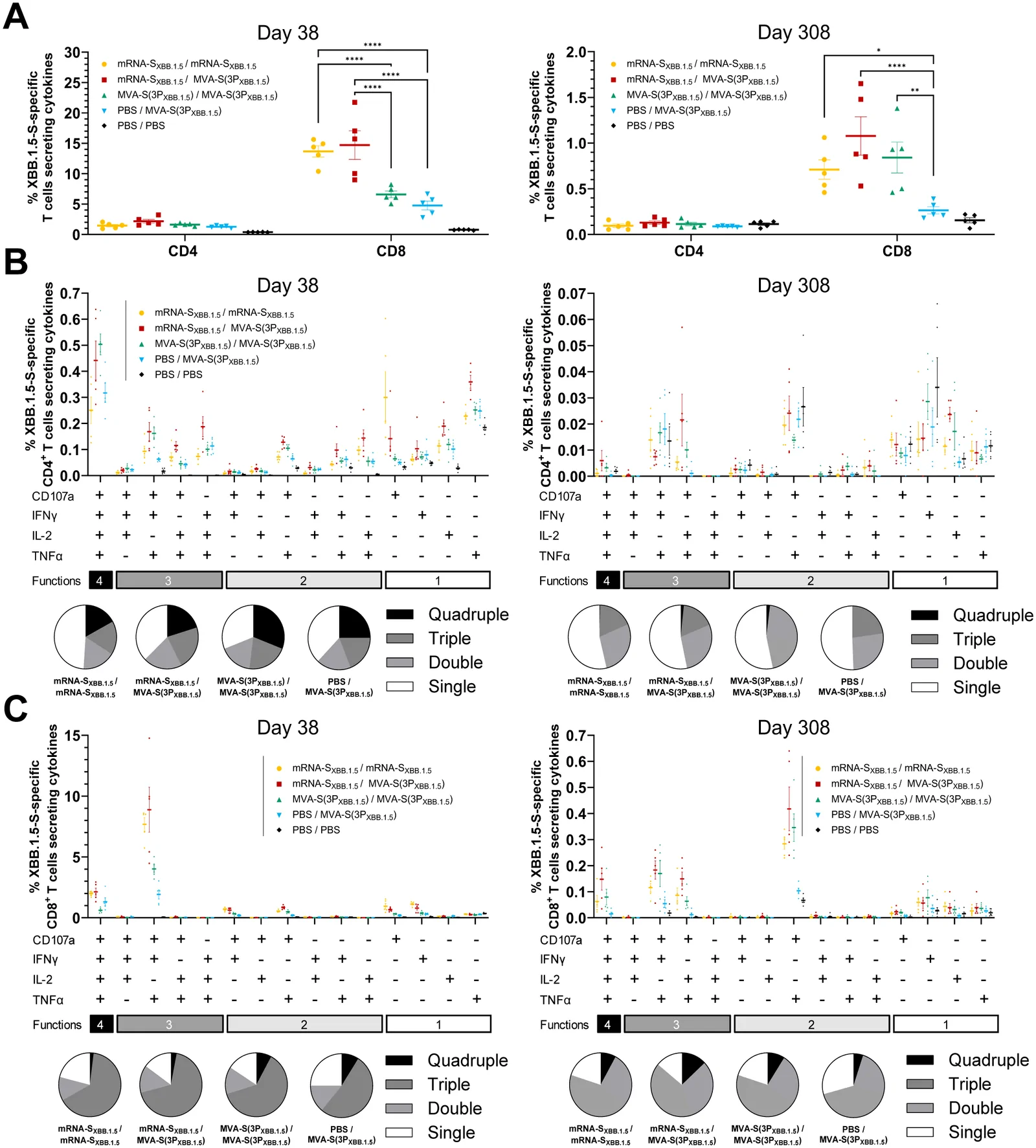
Homologous and heterologous mRNA/MVA vaccination promotes durable SARS-CoV-2 S-specific T-cell responses. **(A)** Magnitude of XBB.1.5 S-specific CD4^+^ and CD8^+^ T-cell responses in splenocytes harvested at days 38 (left panel) and 308 (right panel). Percentages (mean ± SD) of S-specific CD4^+^ or CD8^+^ T cells expressing CD107a and/or producing IFN-γ and/or TNF-α and/or IL-2 are shown. Data are from individual spleen samples from each group. Ordinary two-way ANOVA followed by Tukey’s multiple comparisons test: *p < 0.033; **p < 0.002; ****p<0.0001. **(B, C)** Polyfunctional profiles (based on the expression of CD107a, IFNγ, TNFα, and IL-2) of S-specific CD4^+^ **(B)** and CD8^+^ **(C)** T cells in splenocytes harvested at days 38 (left panels) and 308 (right panels). The response profiles are shown on the x axis, and the percentages of T cells for each vaccination group are shown on the y axis. Pie charts summarize the percentage of S-specific T cells exhibiting one, two, three, or four markers, shown in colour.

At both time points, S-specific T cell responses were predominantly CD8^+^ (**Figure 5A**). At day 38, the mRNA-S_XBB.1._5/mRNA-S_XBB.1.5_ and mRNA-S_XBB.1.5_/MVA-S(3P_XBB.1.5_) groups elicited the highest frequencies of Omicron XBB.1.5 S-specific CD8^+^ T cells (~13-16%), significantly greater than those observed in the MVA-S(3P_XBB.1.5_)/MVA-S(3P_XBB.1.5_) group and in the single-dose MVA-S(3P_XBB.1.5_) group (**Figure 5A**, left panel). In contrast, CD4^+^ T cell responses remained low across all groups (~0-3%) with no significant differences between regimens (**Figure 5A**, left panel). By day 308, the magnitude of CD8^+^ T cell responses underwent a marked contraction, declining to levels below 2% across all immunization regimens, consistent with the transition from an effector to a memory T-cell response (**Figure 5A**, right panel).

Although the magnitude of CD4^+^ T cell responses did not differ significantly among groups, analysis of CD4^+^ T cell polyfunctionality revealed a clear temporal contraction in cytokine co-expression profiles (**Figure 5B**). At day 38, the most frequent population consisted of CD4^+^ S-specific T cells expressing 4 cytokines, with regimens including MVA-S(3P _XBB.1.5_) as a booster inducing higher frequencies of these highly polyfunctional cells. By day 308, CD4^+^ T cell polyfunctionality had markedly declined across all groups, with most cells expressing only one or two cytokines (**Figure 5B**, right panel), indicating a shift toward less complex functional profiles during the memory phase.

Regarding CD8^+^ T cell polyfunctionality, at day 38 the most frequent population consisted of cells expressing CD107a, IFN-γ, and TNF-α, with mRNA-S_XBB.1.5_-primed regimens inducing higher frequencies of these triple-positive cells. Although the overall magnitude of the CD8^+^ T cell response declined by 9 months after the last vaccination, the proportion of S-specific CD8^+^ T cells expressing two or more effector functions remained comparable across the two-dose vaccination groups. In contrast, the single-dose MVA-S(3P _XBB.1.5_) group displayed a higher proportion of monofunctional CD8^+^ T cells, consistent with a less functionally diverse response.

### Homologous and heterologous mRNA/MVA vaccination against SARS-CoV-2 XBB.1.5 confers long-term control of pulmonary replication following SARS-CoV-2 Omicron XBB.1.5 challenge

To evaluate long-term protective efficacy, mice were challenged intranasally with 1 × 10^5^ PFUs of SARS-CoV-2 XBB.1.5 on day 308, corresponding to approximately 9 months after the last vaccination. Mice were monitored for three days post-challenge for weight loss; however, none of the groups, including the unvaccinated PBS/PBS control, exhibited detectable body weight loss (data not shown).

**Figure 6:**
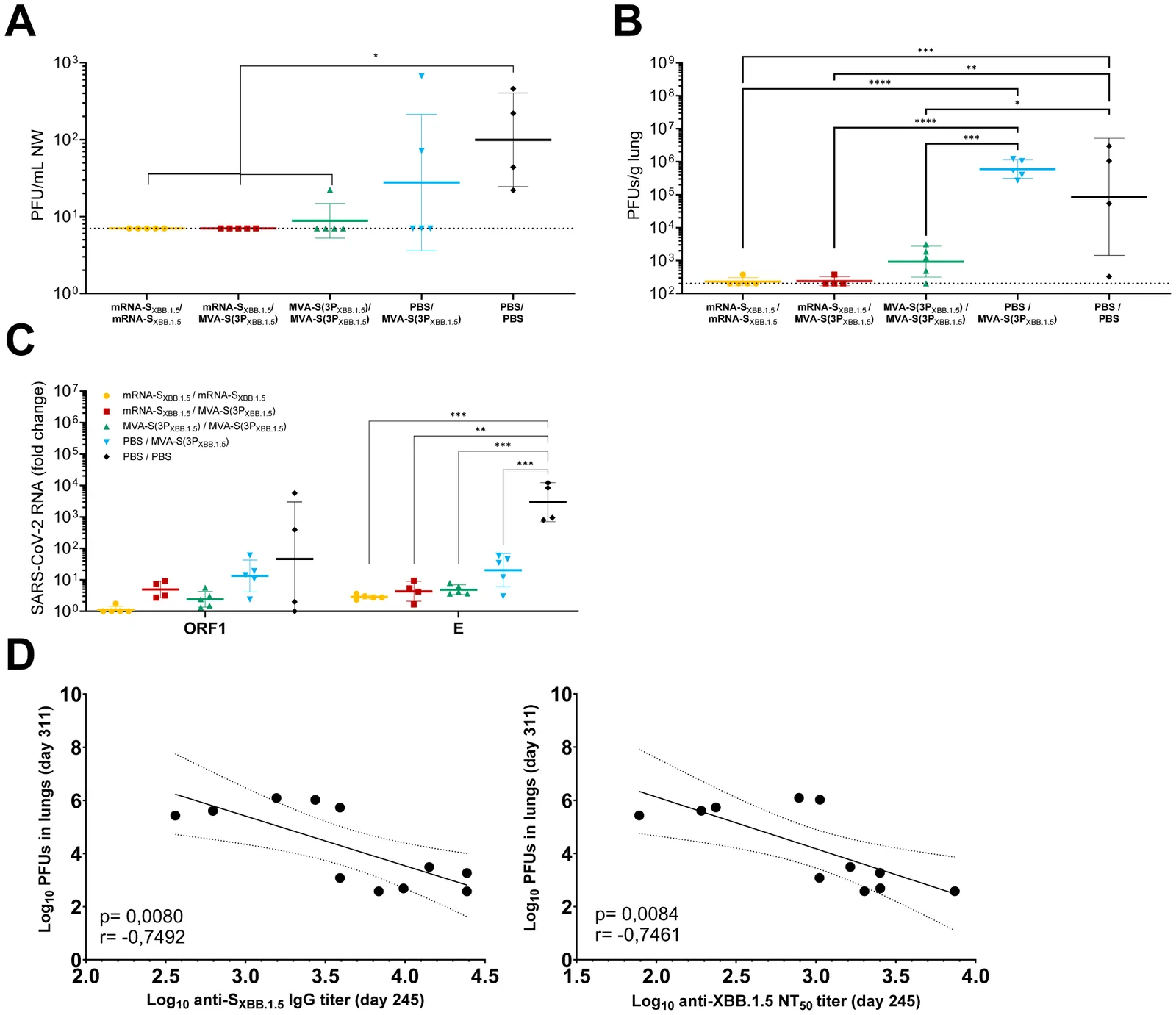
Homologous and heterologous mRNA/MVA vaccination confers long-term protection against SARS-CoV-2 Omicron XBB.1.5. **(A)** Groups of K18-hACE2 transgenic mice (n = 5 per group) were immunized intramuscularly with the prime/boost regimens detailed in the Methods section and in **Figure 2A**. On day 308, animals were intranasally challenged with 1 x 10^5^ PFUs of SARS-CoV-2 Omicron XBB.1.5. At day 3 post-challenge, all mice were sacrificed and nasal washes (NW), lungs, and serum samples were collected. **(A, B)** SARS-CoV-2 infectious virus in NW **(A)** and lungs **(B)** at 3 days post-challenge. The geometric mean ± geometric SD of live virus (PFUs/mL of NW or PFUs/g of lung tissue) from triplicates of each sample for each group is shown. The dotted line represents the limit of detection. Lognormal ordinary one-way ANOVA followed by Tukey’s multiple comparison test: *p < 0.033; **p < 0.002; ***p < 0.0002; ****p<0.0001. **(C)** SARS-CoV-2 genomic ORF1 and subgenomic E RNA levels at 3 days post-challenge. Geometric mean values ± geometric SD are shown. RNA levels were measured by RT-qPCR and are expressed as fold change relative to uninfected mice. Each data point represents the mean of duplicate measurements from individual lung samples. The limit of detection was 1 (10^0^). Ordinary one-way ANOVA followed by Tukey’s multiple comparisons test: **p < 0.002; ***p < 0.0002. **(D)** Correlation between SARS-CoV-2 infectious virus in lung samples and antibody responses. Correlation analysis between SARS-CoV-2 infectious virus in lung samples (log_10_) (day 311) and anti-S Omicron XBB.1.5 IgG titers at day 245 (left panel) or between SARS-CoV-2 infectious virus in lung samples (log_10_) (day 311) and anti-Omicron XBB.1.5 NT_50_ titers at day 245 (right panel). Antibody titers are expressed as log_10_ endpoint titers. Each dot represents an individual mouse. The solid line indicates the linear regression, and dotted lines represent the 95% confidence interval. Pearson correlation coefficients **(r)** and p values are shown.

Infectious virus was quantified in both the upper (nasal wash, NW) and lower respiratory tract (lungs) on day 311 (3 days post-challenge), revealing marked differences among groups. In the upper respiratory tract, low levels of infectious virus were detected even in unvaccinated PBS/PBS control mice. Nevertheless, all two-dose vaccination groups showed a significant reduction in infectious virus titers in NW compared with the unvaccinated PBS/PBS group (**Figure 6A**). In the lungs, all three two-dose vaccination regimens markedly reduced infectious virus levels, with viral titers clustering near the lower LOD and no significant differences observed among them (**Figure 6B**).

Quantification of viral RNA further supported these findings (**Figure 6C**). Analysis of genomic RNA (ORF1), which reflects both residual input virus and total viral RNA burden, showed lower median levels in all two-dose immunization groups compared with the unvaccinated (PBS/PBS) control group, although these differences did not reach statistical significance due to high variability within the control group (**Figure 6C**, left panel). In contrast, analysis of subgenomic RNA, reflecting active viral replication, revealed reduced E gene mRNA levels in all immunized groups, including the single-dose MVA-S(3P_XBB.1.5_) group, compared with unvaccinated PBS/PBS controls (**Figure 6C**, right panel), indicating that two-dose vaccination regimens strongly limited viral replication, whereas a single dose of MVA-S(3P_XBB.1.5_) was associated with a partial reduction in viral replication.

Finally, we assessed whether vaccine-induced antibody responses correlated with control of viral replication (**Figure 6D**). Pre-challenge (day 245) anti-S XBB.1.5 IgG titers and neutralizing antibody titers (NT_50_) against XBB.1.5 both showed significant inverse correlations with infectious viral loads in the lungs (**Figure 6D**), supporting a key role for humoral immunity in controlling viral replication following challenge.

### Homologous and heterologous mRNA/MVA vaccination against SARS-CoV-2 XBB.1.5 is associated with distinct lung transcriptomic profiles following SARS-CoV-2 Omicron XBB.1.5 challenge

To define the global host transcriptional response to SARS-CoV-2 Omicron XBB.1.5 infection and the impact of different homologous and heterologous mRNA- and MVA-based vaccination regimens, we performed RNA-seq analysis on lung samples from challenged K18-hACE2 mice. SARS-CoV-2-challenged mice primed and boosted with PBS (unvaccinated) were used as infection controls, whereas non-vaccinated/non-infected mice served as healthy controls.

**Figure 7:**
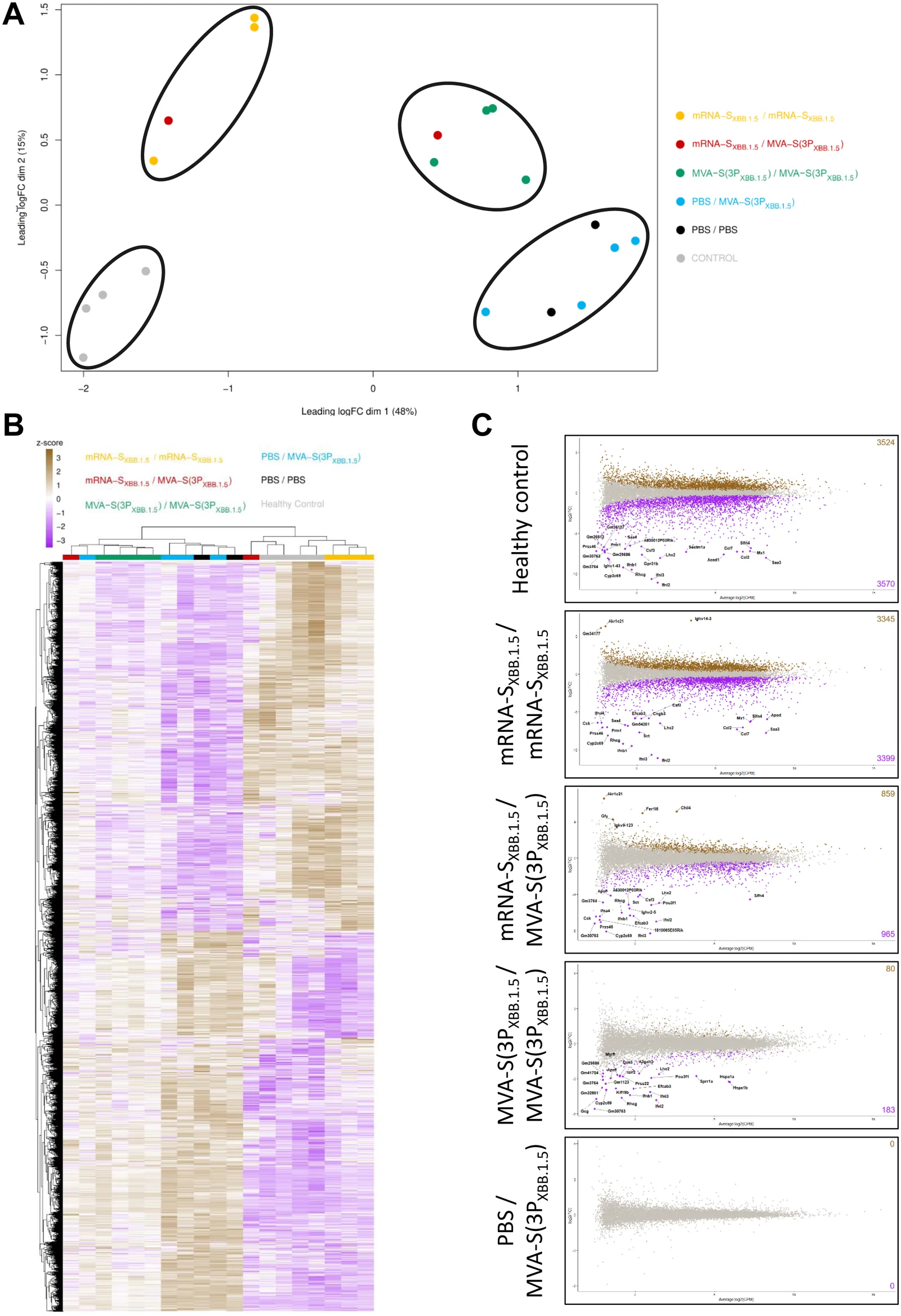
Homologous and heterologous mRNA/MVA vaccination reveals distinct lung transcriptomic profiles after SARS-CoV-2 Omicron XBB.1.5 challenge. **(A)** Multidimensional scaling (MDS) plot of lung transcriptomic profiles showing clustering of samples according to vaccination regimen and infection status. **(B)** Heatmap of differentially expressed genes (DEGs) in lung samples across the different experimental groups. All DEGs identified across all comparisons (n = 8693) are represented. **(C)** Volcano plots showing the DEGs obtained from the 4 immunization groups and healthy controls versus the unvaccinated infection control (PBS/PBS). Only gene symbols from the top 25 DEGs are shown. Data were obtained from RNA-seq analysis of lung samples collected at day 3 post-challenge. Differential expression analysis was performed using edgeR, and genes with a false discovery rate (FDR) q-value < 0.05 were considered significantly differentially expressed.

Multidimensional scaling (MDS) analysis of the lung transcriptomic profiles revealed four distinct clusters (**Figure 7A**). Dimension 1 (dim1), explaining 48% of the total variance, clearly separated healthy control mice from infected PBS/PBS mice. Notably, infected PBS/PBS mice clustered together with mice immunized with a single dose of MVA-S(3P_XBB.1.5_), consistent with the more limited control of pulmonary viral replication observed in this group (**Figure 7A**). Among two-dose regimens, mRNA-S_XBB.1.5_/mRNA-S_XBB.1.5_-immunized mice and one mouse from the heterologous mRNA-S_XBB.1.5_/MVA-S(3P_XBB.1.5_) regimen (with lower pulmonary viral RNA levels) clustered closer to healthy controls along dim1, whereas MVA-S(3P_XBB.1.5_)/MVA-S(3P_XBB.1.5_)-immunized mice and one mouse from the heterologous mRNA-S_XBB.1.5_/MVA-S(3P_XBB.1.5_) regimen (with higher pulmonary viral RNA levels) clustered closer to infection controls along dim1 (**Figure 7A**). Dimension 2 (15% variance) further separated vaccinated groups, indicating additional transcriptional heterogeneity.

These patterns were confirmed by heatmap clustering analysis (**Figure 7B**), where mRNA-S_XBB.1.5_/mRNA-S_XBB.1.5_-immunized mice displayed gene expression profiles more similar to healthy controls, whereas MVA-S(3P_XBB.1.5_)/MVA-S(3P_XBB.1.5_)-immunized mice clustered closer to infected animals (**Figure 7B**). These findings are consistent with the MDS results and indicate that the different vaccination regimens were associated with distinct lung transcriptomic profiles following challenge, despite broadly comparable control of viral replication among the two-dose vaccination groups.

Differential expression (DE) analysis relative to infection controls (PBS/PBS) identified 7,094 differentially expressed genes (DEGs) in healthy controls (3,524 upregulated and 3,570 downregulated), 6,744 in the mRNA-S_XBB.1.5_/mRNA-S_XBB.1.5_ group (3,345 upregulated and 3,399 downregulated), 1,824 in the mRNA-S_XBB.1.5_/MVA-S(3P_XBB.1.5_) group (859 upregulated and 965 downregulated), and 263 in the MVA-S(3P_XBB.1.5_)/MVA-S(3P_XBB.1.5_) group (80 upregulated and 183 downregulated) (**Figure 7C**). Consistent with the MDS and heatmap analyses, no DEGs were detected between single-dose MVA-S(3P_XBB.1.5_) and infection control groups (**Figure 7C**). These results indicate a gradient in lung transcriptional responses across vaccination regimens, with the lung transcriptional profile of mRNA-based vaccinated mice showing the greatest similarity to that of healthy controls.

**Figure 8:**
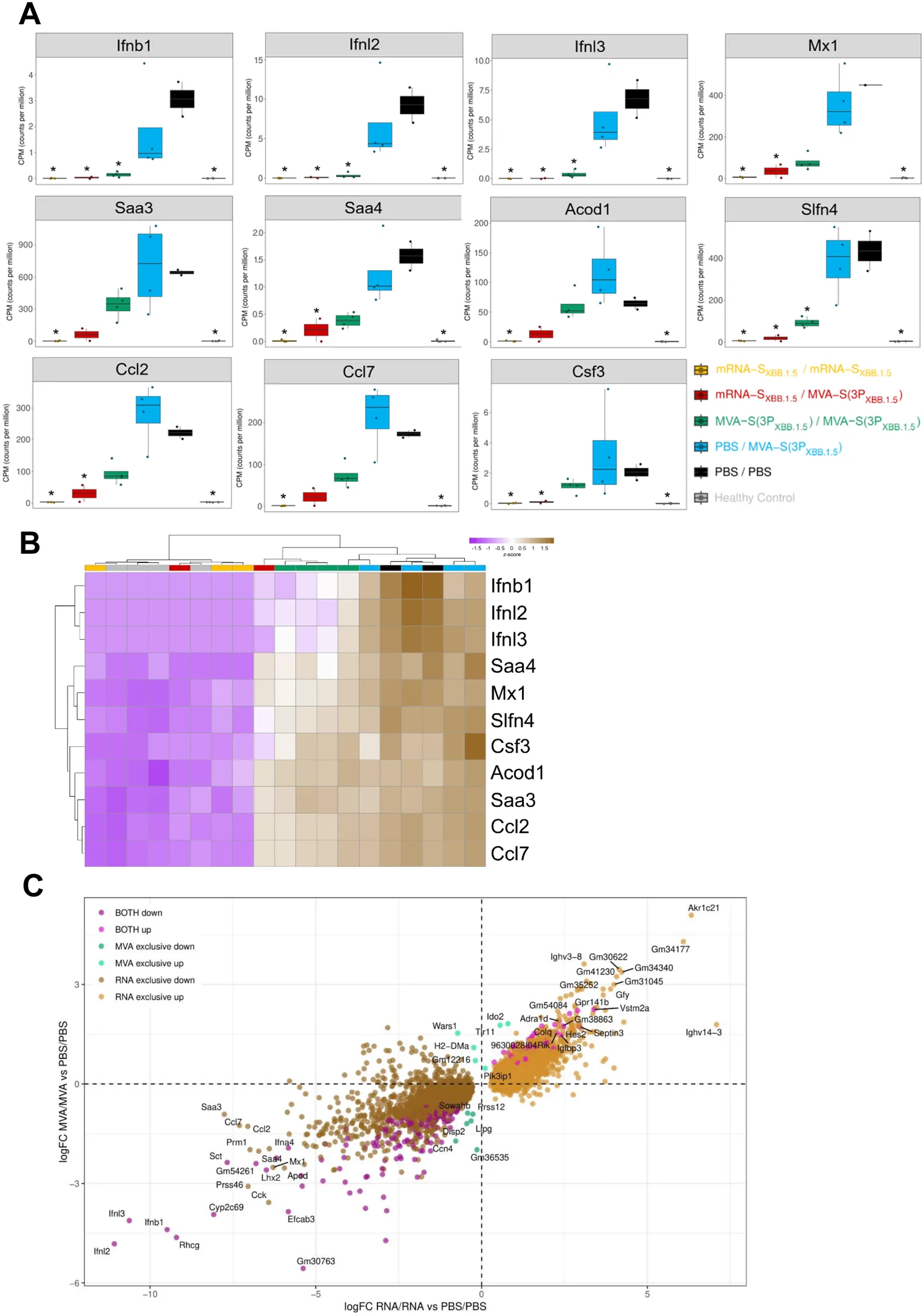
Homologous and heterologous mRNA/MVA vaccination differentially modulates lung inflammatory and immune transcriptional programs after SARS-CoV-2 XBB.1.5 challenge. **(A)** Boxplots showing normalized expression of selected highly abundant genes with robust changes between healthy mice and vaccinated mice compared with unvaccinated infected controls (PBS/PBS). Asterisks indicate significant differences (FDR < 0.05) relative to the PBS/PBS group. **(B)** Heatmap of transcriptional signatures among vaccinated groups, showing differential clustering patterns between homologous and heterologous vaccination regimens. **(C)** Comparative lung transcriptional signatures elicited by homologous mRNA-S_XBB.1.5_ and MVA-S(3P_XBB.1.5_) vaccination after SARS-CoV-2 Omicron XBB.1.5 challenge. Scatter plot showing log_2_ fold changes (logFC) in gene expression in lungs from K18-hACE2 mice vaccinated with mRNA-S_XBB.1.5_/mRNA-S_XBB.1.5_ (x-axis) and MVA-S(3P_XBB.1.5_)/MVA-S(3P_XBB.1.5_) (y-axis) compared with PBS/PBS infection controls at day 3 post-challenge. Each dot represents a differentially expressed gene (DEG); genes commonly upregulated or downregulated by both regimens (BOTH up/down) and genes exclusively modulated in the mRNA- or MVA-vaccinated groups (mRNA exclusive up/down; MVA exclusive up/down) are color-coded as indicated. Labelled genes correspond to the top DEGs in each category ranked by log_2_ fold change (up to 10 genes per category).

DE analysis was performed between healthy animals and the four vaccinated groups versus the non-vaccinated infection control group (PBS/PBS) (**Supplementary Table 1**). At 3 days post-challenge, SARS-CoV-2 Omicron XBB.1.5 infection induced a strong inflammatory and interferon (IFN)-driven transcriptional response in the lungs, characterized by upregulation of type I/III IFNs (Ifnb1, Ifnl2, Ifnl3), IFN-stimulated genes (ISG) (Mx1), acute-phase and immunometabolic genes (Saa3, Saa4, Acod1, Slfn4), and chemokines involved in myeloid cell recruitment (Ccl2, Ccl7, Csf3) compared with healthy controls (**Figure 8A**). In parallel, numerous genes associated with epithelial integrity and tissue-homeostasis were significantly downregulated, although with more modest fold changes than those observed for upregulated genes, indicating a measurable but comparatively less pronounced suppression of normal lung programs and an overall shift toward a transcriptional state consistent with SARS-CoV-2-induced lung inflammation (**Supplementary Table 1**). All two-dose vaccination regimens were associated with reduced expression of IFN-driven and inflammatory genes, with expression levels shifting toward those observed in healthy controls (**Figure 8A**). However, comparative analysis revealed differences in the resulting transcriptional profiles between mRNA-S_XBB.1.5_/mRNA-S_XBB.1.5_- and MVA-S(3P_XBB.1.5_)/MVA-S(3P_XBB.1.5_)-vaccinated mice. The heterologous mRNA-S_XBB.1.5_/MVA-S(3P_XBB.1.5_) group displayed an intermediate phenotype, with one mouse clustering more closely with the mRNA-S_XBB.1.5_/mRNA-S_XBB.1.5_ profile and the other mouse resembling the MVA-S(3P_XBB.1.5_)/MVA-S(3P_XBB.1.5_) profile (**Figure 8B**). To further dissect these differences, we analyzed the top DEGs in each vaccinated group relative to infection controls (PBS/PBS), classifying them as genes exclusively up- or downregulated in the mRNA-S_XBB.1.5_/mRNA-S_XBB.1.5_ group, exclusively regulated in the MVA-S(3P_XBB.1.5_)/MVA-S(3P_XBB.1.5_) group, or commonly up- or downregulated in both vaccination regimens (**Figure 8C**).

Both mRNA-S_XBB.1.5_/mRNA-S_XBB.1.5_ and MVA-S(3P_XBB.1.5_)/MVA-S(3P_XBB.1.5_) regimens shared a transcriptional pattern characterized by reduced expression of type I/III IFN genes (Ifnb1, Ifnl2, Ifnl3) relative to infection controls, consistent with reduced IFN-driven inflammatory responses. However, differences were observed in the genes differentially regulated in each vaccination group. The mRNA-S_XBB.1.5_/mRNA-S_XBB.1.5_ group showed upregulation of immunoglobulin-related genes (Ighv14-3, Ighv1-20, Iglv1, Ighv1-58) together with downregulation of genes associated with acute-phase responses and myeloid-recruitment (Saa3, Ccl7, Ccl2, Prss46, Prm1, Saa4, Cck, Ifna4, Apod, Csf3). In contrast, the MVA-S(3P_XBB.1.5_)/MVA-S(3P_XBB.1.5_) group showed upregulation of genes associated with antigen processing/presentation and innate immune regulation (Ido2, Tlr11, Wars1, H2-DMa, Gm12216, Pik3ip1) together with downregulation of genes associated with tissue remodelling and metabolism (Gm36535, Ccn4, Disp2, Lipg, Prss12, Sowahb).

## Discussion

Our findings are consistent with and extend previous studies demonstrating the immunogenicity and protective efficacy of MVA-based SARS-CoV-2 vaccine candidates using different antigen designs and vaccination strategies. MVA vectors expressing full-length native or prefusion-stabilized S proteins, as well as RBD-based antigens, have elicited robust humoral and cellular immune responses and conferred protection in different preclinical models (5–10, 12–20, 38–40). Moreover, both homologous MVA vaccination and heterologous regimens combining MVA with other vaccine platforms have been shown to induce complementary humoral and cellular immune responses (7, 16). Our study extends these observations by providing a direct longitudinal comparison of homologous mRNA/mRNA, homologous MVA/MVA, and heterologous mRNA/MVA regimens targeting the same XBB.1.5 S antigen, including a clinically approved variant-matched mRNA vaccine as comparator, with immune responses and protective efficacy evaluated up to 9 months after vaccination. In addition, the longitudinal assessment of Fc-mediated antibody functions and post-challenge lung transcriptomic profiles provides complementary insights into the durability and functional characteristics of the immune responses elicited by these vaccination strategies.

Combining MVA-based vaccines with other platforms, such as mRNA or adenoviral vectors (AdV), has proven highly effective in inducing durable and protective humoral and cellular immune responses. A prominent example is the licensed Ebola virus (EBOV) vaccine regimen, which consists of priming with Zabdeno (Ad26.ZEBOV) followed by boosting with Mvabea (MVA-BN-Filo). This heterologous strategy is particularly advantageous because MVA efficiently amplifies both T-cell and antibody responses while remaining largely unaffected by pre-existing anti-vector immunity elicited by AdV-based vaccines (41, 42).

In the context of SARS-CoV-2 vaccination, accumulating evidence indicates that heterologous vaccination strategies promote a more balanced and durable modulation of immune responses than homologous regimens (43, 44). A recognized limitation of mRNA vaccines is the waning of humoral immunity over time; often requiring repeated booster doses to maintain high levels of neutralizing antibodies (45). In this setting, heterologous vaccination approaches may offer advantages by enhancing the magnitude, breadth, and longevity of both humoral and cellular immune responses. Consistent with this, recent studies have shown that heterologous prime/boost regimens, such as mRNA priming followed by AdV boosting, improve the durability of vaccine-induced immunity (46). MVA vectors offer additional advantages as vaccine platforms, including a well-established safety profile and suitability for use in immunocompromised individuals (47). Notably, studies in cancer patients vaccinated against SARS-CoV-2 have shown that MVA-based vaccines can elicit robust humoral and cellular immune responses (48), whereas reduced response rates and a more rapid decline of immune responses have been reported following mRNA vaccination in immunocompromised populations (49).

Clinical data further support the immunogenic potential of MVA-based SARS-CoV-2 vaccines. An MVA vaccine expressing both S and nucleocapsid proteins elicited robust humoral and cellular immune responses without safety concerns (3). In contrast, another MVA-based vaccine expressing a prefusion-stabilized S protein was reported to be less immunogenic than licensed mRNA- and ChAd-based vaccines in SARS-CoV-2–naïve individuals (4), underscoring the importance of antigen design, vaccination regimen, and study population. To date, no clinical trials have evaluated heterologous vaccination strategies combining mRNA priming with MVA boosting against SARS-CoV-2. Nevertheless, recent preclinical studies have demonstrated that heterologous mRNA/MVA regimens confer robust protection against severe SARS-CoV-2 disease and elicit superior immunogenicity compared with homologous regimens in both mice (7) and hamsters (22).

Despite the growing body of evidence supporting the efficacy of mRNA and MVA vaccines, either alone or in heterologous combinations, direct long-term comparisons of these vaccination strategies against highly immune-evasive variants such as Omicron XBB.1.5 remain limited. In the present study, we compared homologous mRNA/mRNA, homologous MVA/MVA, and heterologous mRNA/MVA vaccination strategies using matched XBB.1.5 S antigens and evaluated in the K18-hACE2 mouse model humoral and cellular immune responses, protective efficacy, and lung transcriptomic responses up to 9 months after the last vaccination. We demonstrate that all two-dose vaccination regimens, regardless of platform or prime/boost combination, effectively controlled pulmonary viral replication following SARS-CoV-2 Omicron XBB.1.5 challenge 9 months after the last dose, whereas a single MVA-S(3P_XBB.1.5_) dose was insufficient to achieve comparable control of viral replication. Although protection against pulmonary viral replication was broadly comparable across all two-doses regimens, mRNA-primed strategies tended to maintain higher antibody levels over time, suggesting a modest advantage of mRNA priming for sustaining humoral immunity. Although SARS-CoV-2 XBB.1.5 has been reported to induce severe disease in K18-hACE2 mice, including body-weight loss, severe lung pathology and mortality, these manifestations occur after the peak of pulmonary viral replication (50). Since animals in the present study were euthanized three days after challenge to evaluate viral replication and early immune responses at the time of maximal pulmonary viral burden, the absence of body-weight loss was expected and reflects the selected experimental endpoint rather than reduced pathogenicity of the viral variant.

Although antibody levels in humans are generally considered the best available correlate of protection (51), extrapolation of murine immunogenicity data to predict human vaccine efficacy should be interpreted with caution. In particular, the durability of vaccine-induced immune responses observed in this study cannot be directly be extrapolated to humans, as neutralizing antibody responses following primary COVID-19 vaccination decline more rapidly in immunologically naïve individuals, whereas memory B- and T-cell responses are generally more persistent and can be further enhanced by booster vaccinations and/or SARS-CoV-2 exposure (52). Moreover, unlike the immunologically naïve mice used in this study, most humans have experienced multiple vaccine doses, infection, or both, resulting in substantially different baseline immune landscapes.

Neutralizing antibodies are widely considered the strongest correlate of protection against SARS-CoV-2 (53, 54). Consistent with this, in our study, mice vaccinated with a single dose of MVA-S(3P_XBB.1.5_), which lacked detectable neutralizing antibodies at the time of challenge, exhibited lung viral loads comparable to those of unvaccinated controls. In contrast, two-dose regimens maintained detectable neutralizing activity and were associated with improved control of viral replication. Nevertheless, previous studies in the same mouse model have demonstrated protection against SARS-CoV-2 infection in the absence of detectable neutralizing antibodies (8, 38), indicating that additional immune mechanisms contribute to viral control.

Among these mechanisms, Fc-dependent antibody effector functions are increasingly recognized as important contributors to antiviral immunity (55). These functions, which include phagocytosis, complement activation, and NK cell activation, have been associated with protection against multiple viral infections, including influenza (56), HIV (57, 58), and Ebola (59). In our study, mice vaccinated with two-dose regimens, which were protected upon challenge, exhibited higher antibody-dependent Fc-mediated effector activity, such as phagocytosis, complement deposition, and NK cell activation, than unvaccinated mice that failed to control infection, supporting a role for these mechanisms in protection against SARS-CoV-2 Omicron XBB.1.5.

T-cell responses, particularly CD8^+^ T cells, also play a critical role in protection against SARS-CoV-2 by limiting viral replication, preventing progression to severe disease, and contributing to long-term immunity against viral variants through the secretion of cytotoxic, inflammatory, and regulatory cytokines (60, 61). This arm of cellular immunity is notably resilient to viral evolution, as T cells recognize a broad repertoire of epitopes across multiple viral proteins (60). In the K18-hACE2 mouse model, we observed modest CD4^+^ T-cell responses that declined in magnitude and polyfunctionality over time across all immunization groups. In contrast, mRNA priming induced a significantly greater magnitude of CD8^+^ T-cell responses at the adaptive phase (day 10 after the last vaccination), and although CD8^+^ T-cell magnitude declined by 9 months after the last vaccination and became comparable across all two-dose vaccination regimens, CD8^+^ T-cell polyfunctionality was largely maintained. These findings, together with recent longitudinal data showing that while homologous mRNA vaccination elicits stronger humoral immunity, robust cellular responses induced by heterologous regimens may compensate by balancing protection against breakthrough infection (62), supporting the potential advantages of heterologous vaccination strategies.

Consistent with the experimental design discussed above, SARS-CoV-2 XBB.1.5 challenge did not induce detectable body-weight loss or mortality under the conditions used in this study. Therefore, reductions in pulmonary viral replication and inflammatory responses represent more informative efficacy endpoints than clinical disease in this experimental setting. These characteristics of the XBB.1.5 challenge model should be considered when interpreting the protective efficacy observed in this study.

Beyond the observed control of viral replication, our transcriptomic analysis revealed distinct lung gene expression profiles across vaccination regimens following challenge. Differential expression profiling showed that the different vaccination strategies induced both quantitative and qualitative differences in host gene modulation in the lungs following challenge with the SARS-CoV-2 XBB.1.5 variant. In line with previous work in K18-hACE2 mice, non-human primates, and humans showing that effective vaccination or pre-existing immunity can attenuate infection-induced inflammatory signatures, our findings indicate that vaccination was associated with reduced pulmonary inflammatory responses following challenge. Similar findings were observed in our previous work with the MVA-S vaccine, in which unvaccinated mice challenged 5 weeks after the final dose showed strong activation of innate immune pathways, including neutrophil, IFN, and cytokine signalling, whereas mice receiving two doses of MVA-S displayed lung transcriptional profiles largely restored to levels observed in healthy animals, an effect not observed in animals receiving a single dose (39). Interestingly, a more recent randomized controlled trial also reported attenuation of inflammatory blood RNA signatures in vaccinated individuals compared with placebo recipients following immunization with a recombinant viral vaccine, further supporting the association between effective vaccination and reduced inflammatory transcriptional responses (63). Regarding the differences among vaccination regimens, the mRNA-S_XBB.1.5_/mRNA-S_XBB.1.5_ group was associated with a lung transcriptional profile characterized by immunoglobulin gene upregulation and reduced expression of acute-phase, IFN-I, and myeloid-recruiting genes, whereas MVA-S(3P_XBB.1.5_)/MVA-S(3P_XBB.1.5_) vaccination was associated with differential modulation of genes involved in antigen processing and presentation and innate immune sensing and regulation. The heterologous mRNA-S_XBB.1.5_/MVA-S(3P_XBB.1.5_) regimen displayed an intermediate transcriptional profile. These observations indicate that the different vaccination regimens were associated with distinct patterns of lung gene expression following XBB.1.5 challenge. However, these differences should not be interpreted as definitive platform-specific mechanisms of protection, as post-challenge transcriptional profiles may also be influenced by residual viral burden, the magnitude of the inflammatory response, and the limited sample size analyzed. Further studies will therefore be required to determine the extent to which these transcriptional patterns reflect intrinsic platform-specific immune responses or differences in the post-challenge virological and inflammatory environment.

Several limitations of this study should be considered. First, although the K18-hACE2 mouse model is widely used to evaluate SARS-CoV-2 vaccines and therapeutics, immune responses in mice do not fully recapitulate the complexity of human immunity, and therefore translation of these findings to humans requires caution. In immunologically naïve humans, neutralizing antibody responses following primary COVID-19 vaccination decline substantially over time (64, 65), whereas memory B- and T-cell responses and protection against severe disease are generally more durable (64, 66, 67). Additional booster vaccinations can restore neutralizing antibody responses and improve protection against infection (68). Preclinical studies of MVA-based SARS-CoV-2 vaccines have similarly demonstrated durable humoral and cellular immune responses in mice, including responses maintained for several months after vaccination (13, 40). Accordingly, the approximately 9-month durability of vaccine-induced immunity observed in the present study should be interpreted within the context of this experimental mouse model and should not be directly extrapolated to humans. Moreover, whereas the mice used in this study were immunologically naïve before vaccination, most individuals currently possess complex pre-existing immunity resulting from multiple vaccine doses, previous SARS-CoV-2 infections, or both (69). These differences in baseline immunity and vaccination history should be considered when translating our findings to current human populations. Second, although our study provides a comprehensive evaluation of humoral and cellular immune responses together with post-challenge lung transcriptomic profiling, further validation in additional animal models and clinical studies will be required to confirm these findings. We also acknowledge that the doses selected for the mRNA and MVA vaccine platforms were based on previously established immunogenic regimens in mice and therefore do not represent dose-equivalent formulations. Consequently, direct comparisons between both vaccine platforms should be interpreted with appropriate caution.

Nevertheless, this study has several important strengths. The long follow-up period, with immunogenicity and protective efficacy assessed up to 9 months post-vaccination, represents one of the longest assessments reported for MVA-based SARS-CoV-2 vaccination in mice and provides valuable information on the durability of vaccine-induced immunity. Furthermore, the comprehensive immunological characterization, including antibody magnitude, breadth, Fc-dependent effector functions, T-cell responses, and post-challenge lung transcriptomic profiling, provides an integrated view of the immune mechanisms associated with long-term protection.

Taken together, our findings demonstrate that MVA-based vaccination strategies provide durable humoral and cellular immune responses and enable control of SARS-CoV-2 Omicron XBB.1.5 replication following challenge, comparable to that achieved with mRNA vaccines. These results support MVA as an alternative and complementary platform to mRNA to broaden and diversify vaccine-induced immune responses. By combining distinct immunological programs, mRNA and MVA vaccines may enhance the breadth, durability, and functional quality of immune responses against SARS-CoV-2 and other emerging respiratory viruses.

## Data Availability

The original contributions presented in the study are publicly available. This data can be found here: NCBI Gene Expression Omnibus (GEO), accession number GSE344507.
