# Supplementary material for: Long-term immunity and protection against SARS-CoV-2 XBB.1.5 following homologous and heterologous mRNA and MVA vaccination
Source: Front Immunol. 2026 Aug 31;17:1916109. doi: 10.3389/fimmu.2026.1916109 (PMC13572652; doi:10.3389/fimmu.2026.1916109)
Supplement: Supplementary file 1 [file DataSheet1.pdf]

# Supplementary information

# Long-term immunity and protection against SARS-CoV-2 XBB.1.5 following homologous and heterologous mRNA and MVA vaccination

**Patricia Pérez [1,2,†,*], Gloria Esteso [1,†], Isabel García-García [3], Sara Flores [1], Cristina Sánchez-Corzo [1,2], María A. Noriega [1], José M. Casasnovas [4], Mariano Esteban [1], Juan García-Arriaza [1,2,*]**

[1] Department of Molecular and Cellular Biology, Centro Nacional de Biotecnología (CNB), Consejo Superior de Investigaciones Científicas (CSIC), Madrid, Spain, [2] Centro de Investigación Biomédica en Red de Enfermedades Infecciosas (CIBERINFEC), Madrid, Spain, [3] Department of Genetics, Physiology and Microbiology, Faculty of Biological Sciences, Universidad Complutense de Madrid (UCM), Madrid, Spain, [4] Department of Macromolecular Structures, Centro Nacional de Biotecnología (CNB), Consejo Superior de Investigaciones Científicas (CSIC), Madrid, Spain

[†] These authors have contributed equally to this work.

***Correspondence:**
Patricia Pérez (pperez@cnb.csic.es)
Juan García-Arriaza (jfgarcia@cnb.csic.es)

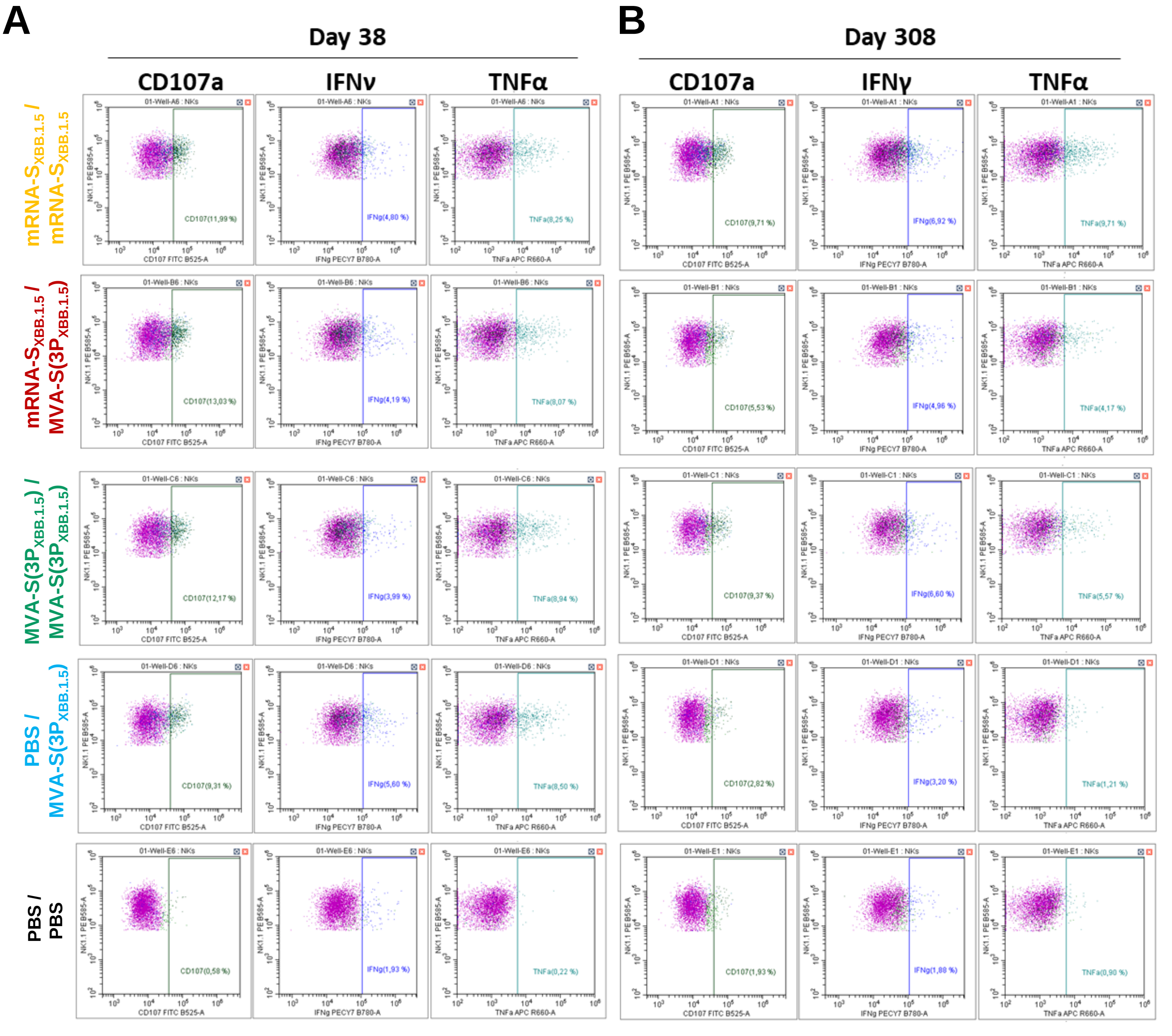


**Supplementary FIGURE 1.** Antibody-dependent NK cell activation (ADNKA) induced by homologous and heterologous mRNA/MVA vaccination against SARS-CoV-2 XBB.1.5. ADNKA against SARS-CoV-2 S protein from Omicron XBB.1.5. Representative flow cytometry plots from serum samples of each group collected at days 38 (A) and 308 (B) are shown, indicating the percentage of NK cells expressing the CD107a degranulation marker or producing IFN-γ or TNF-α.
